# Mechanism of exotic density-wave and beyond-Migdal unconventional superconductivity in kagome metal AV_3_Sb_5_ (A = K, Rb, Cs)

**DOI:** 10.1126/sciadv.abl4108

**Published:** 2022-04-01

**Authors:** Rina Tazai, Youichi Yamakawa, Seiichiro Onari, Hiroshi Kontani

**Affiliations:** Department of Physics, Nagoya University, Furo-cho, Nagoya 464-8602, Japan.

## Abstract

Exotic quantum phase transitions in metals, such as the electronic nematic state, have been discovered one after another and found to be universal now. The emergence of unconventional density-wave (DW) order in frustrated kagome metal AV_3_Sb_5_ and its interplay with exotic superconductivity attract increasing attention. We find that the DW in kagome metal is the bond order, because the sizable intersite attraction is caused by the quantum interference among paramagnons. This mechanism is important in kagome metals because the geometrical frustration prohibits the freezing of paramagnons. In addition, we uncover that moderate bond-order fluctuations mediate sizable pairing glue, and this mechanism gives rise to both singlet s-wave and triplet p-wave superconductivity. Furthermore, characteristic pressure-induced phase transitions in CsV_3_Sb_5_ are naturally understood by the present theory. Thus, both the exotic density wave and the superconductivity in geometrically frustrated kagome metals are explained by the quantum interference mechanism.

## Teaser

Charge density wave and superconductivity in exotic kagome metals are realized by the quantum interference mechanism.

## INTRODUCTION

Rich quantum phase transitions in strongly correlated metals with multiple degrees of freedom and geometrical frustration have been discovered one after another recently (*1*–*4*). To understand such rich phase transitions, an important ingredient is various quantum interference processes among different fluctuations (*5*–*12*). The recent discovery of unconventional density-wave (DW) order and exotic superconductivity in kagome lattice metal AV_3_Sb_5_ (A = K, Rb, Cs) has triggered enormous number of experimental (*13*–*22*) and theoretical (*23*–*28*) researches. In particular, the nontrivial interplay between DW and superconductivity in highly frustrated kagome metals has attracted increasing attention in condensed matter physics.

At ambient pressure, AV_3_Sb_5_ exhibits charge-channel DW order at *T*_DW_ = 78, 94, and 102 K for A = K, Cs, and Rb, respectively (*13*, *14*, *29*, *30*). Below *T*_DW_, a 2 × 2 (inverse) star of David pattern was observed by studies (*31*, *32*). The absence of acoustic phonon anomaly at *T*_DW_ (*33*) would exclude DW states due to strong electron-phonon coupling. As possible electron-correlation–driven DW orders, charge/bond and loop-current (LC) orders (*23*, *25*, *28*, *34*–*36*) have been proposed theoretically, mainly based on the extended Hubbard model with the on-site (*U*) and the nearest-neighbor site (*V*) Coulomb interactions. However, when *V* ≪ *U* due to Thomas-Fermi screening, previous studies predicted strong magnetic instability, in contrast to the small spin fluctuations in AV_3_Sb_5_ at *T*_DW_ (*29*, *30*, *37*).

Below *T*_DW_, exotic superconductivity occurs at *T*_c_ = 1 ∼ 3 K at ambient pressure (*18*, *19*). The finite Hebel-Slichter peak in 1/*T*_1_*T* (*29*) and the absence of the impurity bound-state below *T*_c_ (*38*) indicate the singlet s-wave superconducting (SC) state. On the other hand, the possibilities of triplet pairing state (*39*) and nematic SC state (*40*, *41*) have been reported. In addition, topological states have been discussed intensively (*42*). Under pressure, *T*_DW_ decreases and vanishes at the DW quantum critical point (DW-QCP) at *P* ∼ 2 GPa. For A = Cs, *T*_c_ exhibits a nontrivial double SC dome in the DW phase, and the highest *T*_c_ ( ≲ 10 K) is realized at the DW-QCP (*15*). In addition, theoretical phonon-mediated s-wave *T*_c_ is too low to explain experiments (*24*). Thus, nonphonon SC state due to DW fluctuations (*43*) is naturally expected in AV_3_Sb_5_.

**Fig. 1.**
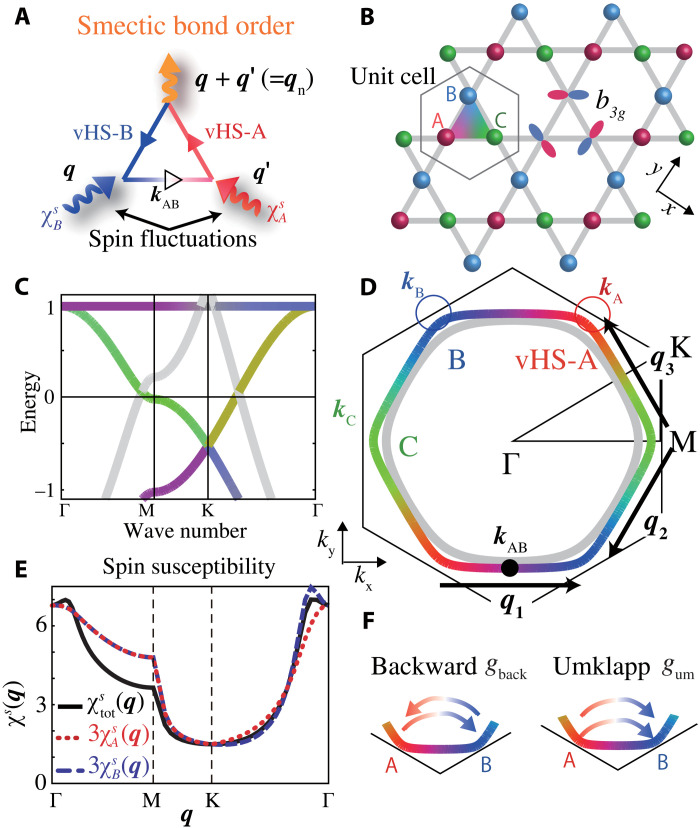
Interference mechanism, FS, and three vHS points in the kagome model. (**A**) Smectic order at wave vector ***q****_n_* = ***q*** + ***q***′ driven by the paramagnon interference mechanism. (**B**) Kagome lattice structure of the vanadium plane. Three *b*_3*g*_ orbitals *A*, *B*, and *C* (and three *b*_2*g*_ orbitals *A*′, *B*′, and C′) are located at A, B, and C sites, respectively. (**C**) Band structure and (**D**) FSs at *n =* 3.8. The outer (inner) FS is composed of *b*_3*g*_ (*b*_2*g*_) orbitals. Three vHS points ***k***_A_, ***k***_B_, and ***k***_C_ are respectively composed of *A* (red), *B* (blue), and *C* (green) orbitals. The inter-vHS nesting vectors *q_n_* (*n* = 1, 2, 3) are shown. All *b*_2*g*_ orbitals are expressed as gray color. (**E**) $\chi_{A}^{s}(q)$, $\chi_{B}^{s}(q)$, and $\chi_{\text{tot}}^{s}(q)$ in the RPA. (**F**) Backward and umklapp scattering between different vHS points. These processes are caused by paramagnon interference mechanism. (See Fig. 3 for details.)

The current central issues would be summarized as (i) origin of the DW state and its driving mechanism, (ii) mechanism of nonphonon SC state, and (iii) interplay between DW and superconductivity. Such nontrivial phase transitions would be naturally explained in terms of the quantum interference mechanism. The interference among spin fluctuations (*5*, *6*, *9*, *10*, *44*, *45*) (at wave vectors ***q*** and ***q***′) give rise to unconventional DW at ***q*** + ***q***′, which is shown in Fig. 1A. This mechanism has been applied to explain the orbital/bond orders in various metals (*4*, *46*–*49*). It is meaningful to investigate the role of the paramagnon interference in kagome metals because the geometrical frustration prohibits the freezing of paramagnons. Its lattice structure, band dispersion, and Fermi surface (FS) with three van Hove singularity (vHS) points are shown in Fig. 1 (B to D, respectively).

Here, we present a unified explanation for the DW order and exotic SC state in geometrically frustrated kagome metal AV_3_Sb_5_ that is away from the magnetic criticality, by focusing on the beyond-mean-field electron correlations. The DW is identified as the “intersublattice bond order” that preserves the time-reversal symmetry. It originates from the paramagnon interference mechanism that provides sizable intersublattice backward and umklapp scattering. In addition, we uncover that the smectic DW fluctuations induce sizable “beyond-Migdal” pairing interaction that leads to the singlet s-wave SC state. The triplet p-wave state also appears when spin and DW fluctuations coexist. The origins of the star of David order, the exotic superconductivity, and the strong interplay among them are uniquely explained on the basis of the quantum interference mechanism. This mechanism has been overlooked previously.

In Discussion, we study the *P-T* phase diagram and the impurity effect on the SC states. The commensurate-incommensurate (C-IC) DW transition is obtained at 1 GPa based on the realistic model constructed by the first-principles study. On the basis of this C-IC transition scenario, we put the following theoretical predictions (i to iv): (i) For 0 ≤ *P* < 1 GPa, the commensurate DW emerges. (ii) For *P* > 1 GPa, the DW state turns to be incommensurate due to the pressure-induced self-doping on the *b*_3*g*_-orbital FS (about 1.5%). (iii) As the highest *T*_c_ dome at *P* ∼ 2 GPa, anisotropic s-wave SC state is realized by the bond-order fluctuations. (iv) In another SC dome at *P* ∼ 0.7 GPa, both s- and p-wave states can emerge because the spin and bond-order fluctuations would be comparable. Thus, impurity-induced p-wave–to–s-wave transition may occur. The present key findings will stimulate future experiments on AV_3_Sb_5_.

## RESULTS

### Band structure with three vHS points

We analyze the following six orbital kagome lattice Hubbard model introduced in (*25*). It is composed of three *b*_3*g*_ orbitals (*A*, *B*, and *C*) and three *b*_2*g*_ orbitals (*A*′, *B*′, *C*′). Orbitals *A* and *A*′ are located at A site, for instance. The kinetic term in ***k***-space is given as

$$H_{0}=\sum_{k,l,m,\sigma }\epsilon_{\mathrm{lm}}(k)c_{k,l,\sigma }^{†}c_{k,m,\sigma }$$ (1)

where *l*, *m* = *A*, *B*, *C*, *A*′, *B*′, *C*′. Here, the unit of energy (in Coulomb interaction, hopping integral, and temperature) is electron volts. The nearest-neighbor hopping integrals are *t*_*b*3*g*_ = 0.5, *t*_*b*2*g*_ = 1, and *t*_*b*3*g*, *b*2*g*_ = 0.002, and the on-site energies are *E*_*b*3*g*_ = −0.055 and *E*_*b*2*g*_ = 2.17 (*25*). In the numerical study, it is convenient to analyze the six-orbital triangular lattice model in fig. S1 in section SA, which is completely equivalent to the kagome metal in Fig. 1B. In the *b*_3*g*_-orbital band shown in Fig. 1D, each vHS point (A, B, and C) is composed of pure orbital (*A*, *B,* and *C*), while the point ***k***_AB_ = (***k***_A_ + ***k***_B_)/2 is composed of orbitals *A* and *B*. The present *b*_3*g*_-orbital FS in the vicinity of three vHS points, on which the pseudogap opens below *T*_DW_ (*50*–*52*), captures the observed FS well (*31*, *53*, *54*).

Next, we introduce the “on-site Coulomb interaction” term *H_U_*. It is composed of the intra- (inter-) orbital interaction *U* (*U*′), and the exchange interaction *J* = (*U* − *U*′)/2. Below, we fix the ratio *J*/*U* = 0.1. The 4 × 4 matrix expression of on-site Coulomb interaction at each site, ${\hat{U}}^{s(c)}$ for spin (charge) channel, is explained in section SA.

In the mean-field-level approximation, the spin instability is the most prominent because of the largest interaction *U*. Figure 1E exhibits the intra-*b*_3*g*_–orbital static (ω = 0) spin susceptibilities $\chi_{A}^{s}(q)\equiv \chi_{\mathrm{AA},\mathrm{AA}}^{s}(q)$ and $\chi_{\text{tot}}^{s}(q)=\sum_{m}^{A,B,C}\chi_{m}^{s}(q)$ in the random phase approximation (RPA) at *U* = 1.26 (α*_S_* = 0.80 at *T* = 0.02). In the RPA, ${\hat{\chi }}^{s}(q)={\hat{\chi }}^{0}(q){\left(\hat{1}-{\hat{\Gamma }}^{s}{\hat{\chi }}^{0}(q)\right)}^{-1}$, where ${\hat{\chi }}^{0}(q)$ is the irreducible susceptibility matrix and *q* ≡ (***q***, ω*_l_* = 2π*Tl*). The spin Stoner factor α*_S_* is the maximum eigenvalue of ${\hat{\Gamma }}^{s}{\hat{\chi }}^{0}(q)$, and magnetic order appears when α*_S_* = 1. Thus, intraorbital spin susceptibility gets enhanced at ***q*** ≈ **0** in the present kagome model. [Note that $\chi_{A}^{s}(q_{1})$ is small because orbitals *A* and *B* correspond to different sites, referred to as the sublattice interference (*34*). In addition, $\chi_{\mathrm{AA},\mathrm{BB}}^{s},\chi_{A\prime }^{s}$ is much smaller than $\chi_{A}^{s}$.]

### Bond order derived from DW equation

Nonmagnetic DW orders cannot be explained in the RPA unless large nearest-neighbor Coulomb interaction *V* (*V* > 0.5*U*) exists. However, beyond-RPA nonlocal correlations, called the vertex corrections (VCs), can induce various DW orders even for *V* = 0 (*5*, *6*, *9*, *10*, *44*). To consider the VCs due to the paramagnon interference in Fig. 1A, which causes the nematicity in Fe-based and cuprate superconductors, we use the linearized DW equation (*10*, *47*)

$$\begin{array}{cc} \lambda_{q}f_{q}^{L}(k) & =-\frac{T}{N}\sum_{p,M_{1},M_{2}}I_{q}^{L,M_{1}}(k,p)\\ & \times {\left\{G(p)G(p+q)\right\}}^{M_{1},M_{2}}f_{q}^{M_{2}}(p) \end{array}$$ (2)

where $I_{q}^{L,M}(k,p)$ is the “electron-hole pairing interaction”, *k* ≡ (***k***, ϵ*_n_*) and *p* ≡ (***p***, ϵ*_m_*) (ϵ*_n_*, ϵ*_m_* are fermion Matsubara frequencies). *L* ≡ (*l*, *l*′) and *M_i_* represent the pair of *d*-orbital indices *A*, *B*, *C*, *A*′, *B*′, *C*′. λ*_q_* is the eigenvalue that represents the instability of the DW at wave vector ***q***, and ${\text{max}}_{q}\{\lambda_{q}\}$ reaches unity at *T* = *T*_DW_. $f_{q}^{L}(k)$ is the Hermite form factor that is proportional to the particle-hole (p-h) condensation $\sum_{\sigma }\langle c_{k+q,l,\sigma }^{†}c_{k,l\prime ,\sigma }\rangle$ or, equivalently, the symmetry breaking component in the self-energy.

It is important to use the appropriate kernel function $I_{q}^{L,M}$, which is given as δ^2^Φ_LW_/δ*G*_*l*^′^*l*_(*k*)δ*G*_*mm*^′^_(*p*) at ***q*** = **0** in the conserving approximation scheme (*44*, *55*), where Φ_LW_ is the Luttinger-Ward function introduced in Materials and Methods. If we apply the bare interaction to $I_{q}^{L,M}$ that corresponds to RPA (*55*), the relation λ*_q_* > α*_S_* cannot be realized when *H_U_* is local. Thus, higher-order corrections are indispensable.

Here, we apply the one-loop approximation for Φ_LW_ (*6*, *44*). Then, $I_{q}^{L,M}$ is composed of one single-magnon exchange Maki-Thompson (MT) term and two double-magnon interference Aslamazov-Larkin (AL) terms. Their diagrammatic and analytic expressions are explained in Materials and Methods. Because of the AL terms, the nonmagnetic nematic order in FeSe is naturally reproduced even if spin fluctuations are very weak (*6*). The importance of AL terms was verified by the functional renormalization group (fRG) study with constrained-RPA in which higher-order parquet VCs are produced in an unbiased way for several Hubbard models (*4*, *7*, *46*). Later, we see that the AL diagrams induce the backward and umklapp scattering shown in Fig. 1F, and they mediate the p-h condensation at the inter-vHS nesting vector ***q***_1_ = ***k***_B_ − ***k***_A_.

**Fig. 2.**
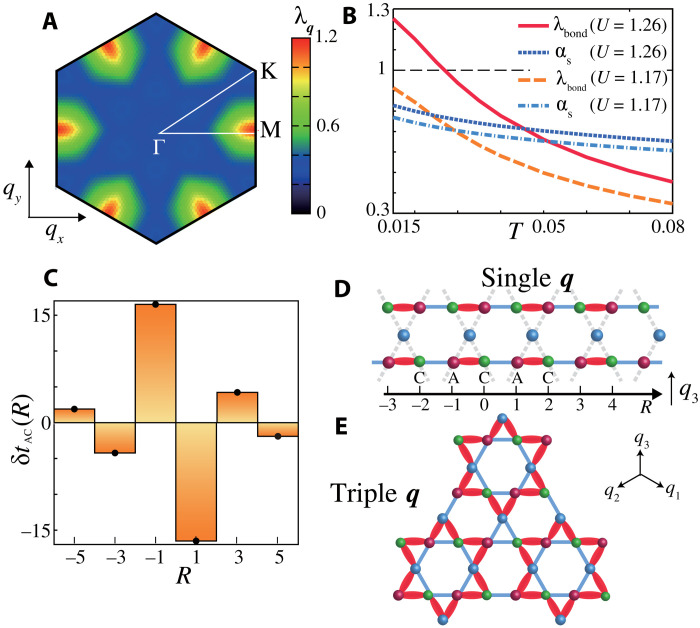
Bond-order solution derived from DW equation. (**A**) Obtained ***q*** dependence of the eigenvalue λ*_q_* at *n =* 3.8 (*T* = 0.02 and α_S_ = 0.80). λ*_q_* shows peaks at ***q***_***n***_ (*n* = 1, 2, 3), consistently with experiments in AV_3_Sb_5_. (**B**) *T* dependences of λ_bond_ and α*_S_* at *U* = 1.26 and 1.17. The DW susceptibility [$\chi_{f}^{c}(q_{n})\propto 1/(1-\lambda_{\text{bond}})$] increases as *T* → *T*_DW_ ≈ 0.025, whereas magnetic susceptibility [χ*^s^*(0) ∝ 1/(1 − α*_S_*)] is almost constant. (**C**) Modulation of hipping integrals δ*t_AC_*(*Ra*_AC_) for ***q = q***_**3**_ along the A-C direction (arbitrary unit). Its schematic picture at wave vector ***q***_**3**_ is shown in (**D**). (**E**) Expected triple-***q*** star of David bond order.

Figure 2A exhibits the obtained ***q*** dependence of the eigenvalue λ_***q***_ at *n* = 3.8 (*T* = 0.02 and α*_S_* = 0.80). The obtained peak position at ***q****_n_* (*n* = 1,2,3) is consistent with experiments in AV_3_Sb_5_. The *T* dependences of λ_bond_ ≡ λ*_q_n__* and α*_S_* are shown in Fig. 2B. The DW susceptibility [$\chi_{f}^{c}(q_{n})\propto 1/(1-\lambda_{\text{bond}})]$ increases as *T* → *T*_DW_ ≈ 0.025, whereas the increment of ferromagnetic susceptibility [χ*^s^*(0) ∝ 1/(1 − α*_S_*)] is small. Then, what order parameter is obtained? To answer this question, we perform the Fourier transform of the form factor

$$\delta t_{\mathrm{lm}}(r)=\frac{1}{N}\sum_{k}f_{q_{n}}^{\mathrm{lm}}(k)e^{ir\cdot k}$$ (3)

Then, δ*t_lm_*(***r****_i_* − ***r****_j_*) cos (***q****_n_* · ***r****_i_* + θ) represents the modulation of the hopping integral between ***r****_i_* and ***r****_j_*, where *r_i_* represents the center of a unit-cell *i* in real space, and θ is a phase factor. The bond order preserves the time-reversal symmetry because it satisfies the relation δ*t_lm_*(***r***) = δ*t_ml_*(−***r***) = real. [Note that the current order is δ*t_lm_*(***r***) = −δ*t_ml_*(−***r***) = imaginary.] Figure 2C represents the obtained form factor δ*t_AC_*(*r*) for ***q*** = ***q***_3_ along the A-C direction, where ***r*** = *Ra*_AC_ with odd number *R*. The obtained solution is a bond order because the relation δ*t_CA_*(***r***) = δ*t_AC_*( − ***r***) is verified. The relation δ*t_AA_*(***r***) = δ*t_CC_*(***r***) = 0 holds in this bond-order solution.

To summarize, we obtain the single-***q*** smectic bond order depicted in Fig. 2D. In section SB, we perform the DW equation analysis for *n* = 3.6 and 3.7 and obtain very similar results to Fig. 2. Thus, the robustness of the bond order is confirmed, irrespective of the Lifshitz transition at *n* ≈ 3.71. In the triple-***q*** state in which three bond orders with ***q***_1_, ***q***_2_, ***q***_3_ coexist, a star of David bond order in Fig. 2E appears. Figure S4 in section SC shows the unfolded FS under the triple-***q*** order below *T*_DW_. In the present model, triple-***q*** order is expected to emerge because the momentum conservation ***q***_1_ + ***q***_2_ + ***q***_3_ = **0** gives rise to the third-order Ginzburg-Landau (GL) free energy *F*^(3)^ = *b*ϕ_1_ϕ_2_ϕ_2_, where ϕ*_n_* is real-order parameter for ***q*** = ***q****_n_* bond order (*n* = 1 − 3) (*48*). Here, $\phi_{n}{\hat{f}}_{q_{n}}(k)$ is the bond-order function, where ${\hat{f}}_{q_{n}}(k)$ is the normalized dimensionless form factor given by the linearized DW equation. A more detailed explanation is given in section SD.

**Fig. 3.**
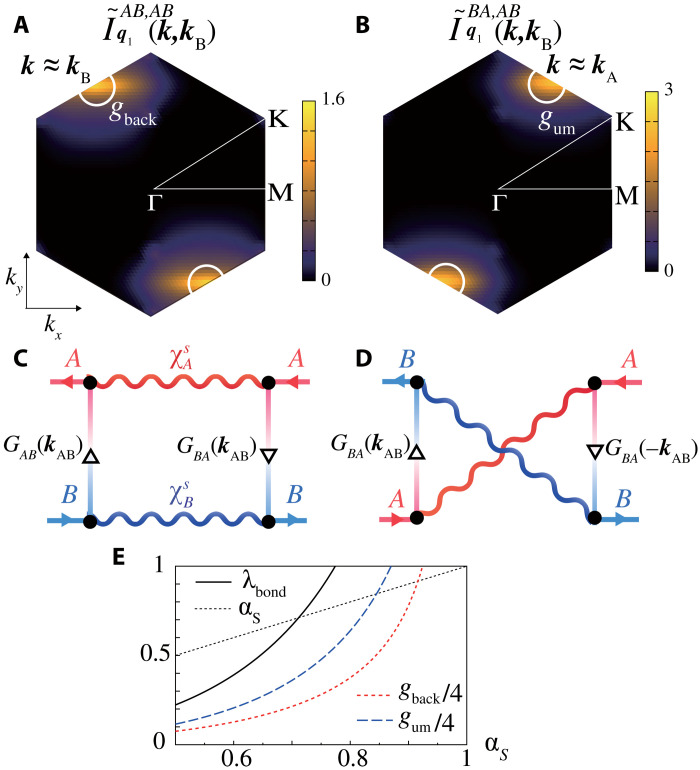
Origin of backward and umklapp scatterings that cause bond-order and SC states. Kernel function in the DW equation with orbital weights at the lowest Matsubara frequency: (**A**) ${\tilde{I}}_{q_{1}}^{\mathrm{AB},\mathrm{AB}}(k,k_{B})$ and (**B**) ${\tilde{I}}_{q_{1}}^{\mathrm{AB},\mathrm{AB}}(k,k_{B})$ for α*_S_* = 0.80. In the kernel function, the outer momenta and sublattices are explained in Materials and Methods. The former at *k* = *k*_B_ and the latter at *k* = *k*_A_ give *g*_back_ and *g*_um_, respectively. Both scatterings contribute to the bond-order formation. (**C**) AL-VC with p-h pair and (**D**) that with p-p pair. The former (latter) gives large *g*_back_ (*g*_um_). (**E**) λ_bond_, *g*_back_, and *g*_um_ as functions of α*_S_* at *T* = 0.02.

We stress that the bond order originates from the intersublattice VC in the kernel function *I* in Eq. 2 [within the RPA, *I* ( = −*U*) is an intrasublattice function]. The dominant form factor at wave vector ***q*** = ***q***_1_, $f_{q_{1}}^{\mathrm{lm}}(k)$, is given by (*lm*) = (*AB*) and (*BA*). To understand its origin, we examine the kernel function at the lowest Matsubara frequency, multiplied with the *b*_3*g*_-orbital weight (*A*, *B*, or *C*) on two conduction bands at four outer points, $\tilde{I}$. Results are shown in Fig. 3 (A) ${\tilde{I}}_{q_{1}}^{\mathrm{AB},\mathrm{AB}}(k,k_{B})$ and (B) ${\tilde{I}}_{q_{1}}^{\mathrm{BA},\mathrm{AB}}(k,k_{B})$, at *T* = 0.02 and α*_S_* = 0.80. They are obtained in the triangular lattice model in fig. S1 that is equivalent to the kagome metal. We see the strong developments of Fig. 3 (A) $g_{\text{back}}\equiv {\tilde{I}}_{q_{1}}^{\mathrm{AB},\mathrm{AB}}(k_{B},k_{B})$ and (B) $g_{\text{um}}\equiv {\tilde{I}}_{q_{1}}^{\mathrm{BA},\mathrm{AB}}(k_{A},k_{B})$, which correspond to the backward and umklapp scattering in Fig. 1F. (We note that the relation ***k***_A_ + ***q***_1_ = ***k***_B_ and four outer momenta and sublattices of *I* are explained in Materials and Methods.) Both scatterings contribute to the bond-order formation, as we clearly explain based on a simple two vHS model in section SB. Microscopic origin of large *g*_back_ [*g*_um_] is the AL-VC with p-h [particle-particle (p-p)] pair shown in Fig. 3 (C and D), because of the relation $\chi_{A}^{s},\chi_{B}^{s}\gg |\chi_{\mathrm{AA},\mathrm{BB}}^{s}|$. They are included as AL1 and AL2 in the kernel function *I*; see Materials and Methods.

In Fig. 3E, we display the increment of λ_bond_, *g*_back_, and *g*_um_ with α*_S_* ( ∝ *U*) at *T* = 0.02. (The relation λ_bond_ ∝ *g*_back_ + *g*_um_ holds, as we explain in section SB.) When α*_S_* = 0.75, then λ_bond_ ≈ 0.88, *g*_um_ ≈ 2 and *g*_back_ ≈ 1, respectively. Thus, both *g*_um_ and *g*_back_ are comparable or larger than *U* due to the quantum interference mechanism in Fig. 3 (C and D) in which the interorbital Green function *G_AB_*(*k*) is substantial. As understood from Fig. 1B, *G_AB_*(*k*) is large at ***k*** ∼ ***k***_AB_, while it vanishes at ***k*** = ***k***_A_ and ***k***_B_. Therefore, the FS portion away from vHS points is indispensable in deriving the smectic order.

### Unconventional superconductivity

**Fig. 4.**
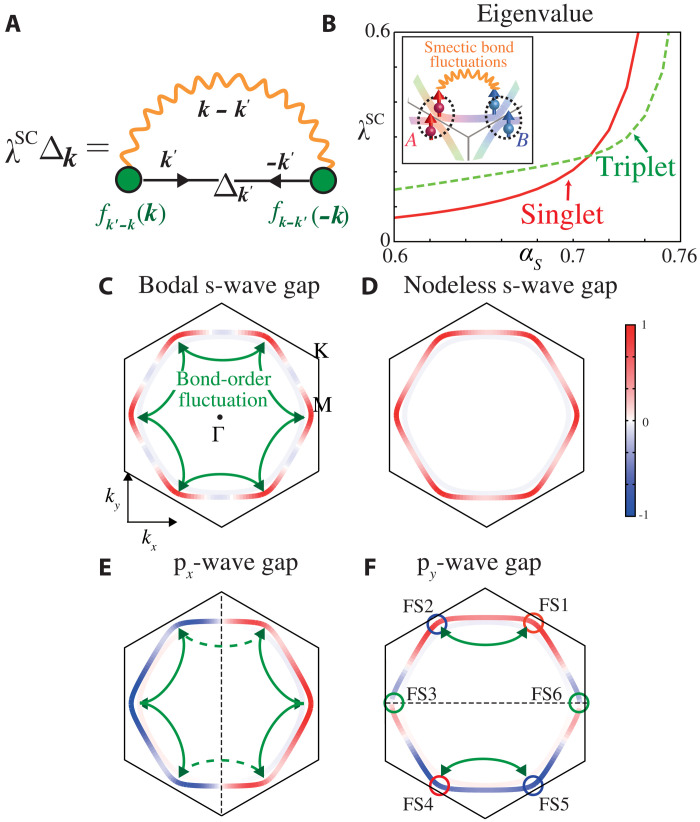
Unconventional SC states due to bond-order fluctuation “beyond-Migdal” pairing glue. (**A**) Pairing gap equation due to bond-order fluctuations. The form factor *f* gives the nonlocal (beyond-Migdal) electron-boson coupling function. (**B**) Obtained eigenvalues of gap equation for the singlet s-wave (*A*_1*g*_) and the triplet p-wave (*E*_1*u*_) states. Obtained gap functions: (**C**) nodal s-wave state (α*_S_* = 0.75), (**D**) nodeless s-wave state (α*_S_* = 0.76), and (**E** and **F**) (p*_x_*, p*_y_*)-wave state (α*_S_* = 0.70). Green full (broken) arrow lines represent the smectic fluctuations between vHS points with the same (opposite) sign gap functions.

Last, we study the unconventional superconductivity mediated by bond-order fluctuations. Here, we solve the following linearized SC gap equation on the FSs

$$\lambda^{\text{SC}}\Delta_{k}(\epsilon_{n})=\frac{\pi T}{{\left(2\pi \right)}^{2}}\sum_{\epsilon_{m}}\;\oint_{\text{FSs}}\frac{dk\prime }{v_{k\prime }}\frac{\Delta_{k\prime }(\epsilon_{m})}{|\epsilon_{m}|}V_{s(t)}^{\text{SC}}(k,k\prime )$$ (4)

where Δ_***k***_(ϵ_n_) is the gap function on FSs, and *v_k_* is the Fermi velocity. The eigenvalue λ^SC^ reaches unity at *T* = *T*_c_. The diagrammatic expression of Eq. 4 is given in Fig. 4A. The form factor represents the “nonlocal” electron-boson coupling function that is a part of the beyond-Migdal effects. $V_{s/t}^{\text{SC}}$ is the singlet/triplet pairing interaction in the band basis, due to the triple-***q*** bond-order fluctuations (*V*_bond_) and spin fluctuations ($\frac{3}{2}U^{2}\chi^{s}$) derived in section SE. Here, *V*_bond_ for ***k***′ − ***k*** ≈ ***q***_1_ is given as

$$\frac{1}{2}\frac{g_{\mathrm{um}}{\bar{f}}_{q_{1}}(k){\bar{f}}_{q_{1}}{\left(-k^{\prime }\right)}^{*}}{1-\lambda_{\text{bond}}}\frac{1}{1+\xi^{2}{\left(q_{1}-(k^{\prime }-k)\right)}^{2}}$$ (5)

where ${\bar{f}}_{q}(k)$ is the Hermite form factor in the band basis, and $|{\bar{f}}_{q_{1}}(k_{A})|=1$. Both λ_bond_ and *g*_um_ are already obtained in Fig. 3E, and the numerator of Eq. 5 on outer FS is given in fig. S6 in section SE.

Figure 4B shows the obtained λ^SC^ at *T* = 0.02 and ξ = 1.0, where the s-wave singlet state appears when α*_S_* ≳ 0.7 and λ_bond_ > α*_S_*. Figure 4 (C and D) exhibits the obtained nodal s-wave gap function at α*_S_* = 0.75 (λ_bond_ = 0.88) and nodeless s-wave one at α*_S_* = 0.76 (λ_bond_ = 0.92), respectively. On the other hand, (p*_x_*, p*_y_*)-wave gap functions obtained at α*_S_* = 0.70 are shown in Fig. 4 (E and F). Note that the obtained SC gap on inner FS made of *b*_2*g*_ orbital is very small, while it can be large owing to (for instance) finite interband electron-phonon interaction.

Here, we discuss the origin of the s/p-wave SC state. Triple-***q*** bond-order fluctuations work as attraction between FS*i* and FS(*i* + 1), where FS*i* (*i* = 1 ∼ 6) is the FS portion around vHS points shown in Fig. 4F. Therefore, six pairs shown by green full arrows contribute to the s-wave state in Fig. 4C. In contrast, only two pairs contribute to the p*_y_*-wave state in Fig. 4F. [In the p*_x_*-wave state in Fig. 4E, four pairs (two pairs) give a positive (negative) contribution.] Therefore, the s-wave state is obtained for α*_S_* ≳ 0.7, where λ_bond_ exceeds α*_S_*. In contrast, the p-wave state is obtained for α*_S_* ≲ 0.7, because weak ferrospin fluctuations favor (destroy) the triplet (singlet) pairing. Thus, the present spin + bond-order fluctuation mechanism leads to rich s- and p-wave states. Possible SC states in the *P-T* phase diagram in CsV_3_Sb_5_ will be discussed in Discussion.

The nodal gap structure shown in Fig. 4C is obtained in the case of α*_S_* = 0.75 (*U* = 1.18). We verified that the nodal s-wave gap structure emerges away from the vHS points so as to minimize the “depairing effect by moderately ***k***-dependent repulsion by weak spin fluctuations,” which are shown in Fig. 1E. On the other hand, the nodeless s-wave state is realized when α_S_ = 0.76, as shown in Fig. 4D. The reason is that the attraction due to bond-order susceptibility [∝1/(1 − λ_bond_)] is strongly enhanced for α*_S_* ≳ 0.7 as recognized in Fig. 3E, and therefore, the reduction of depairing due to nodal structure becomes unnecessary.

To summarize, large attraction between different vHS points is induced by the bond-order fluctuations due to the paramagnon interference process. In contrast, the repulsion between different vHS points due to spin fluctuations is small, by reflecting the fact that the vHS points ***k****_A_*, ***k****_B_*, and ***k****_C_* are respectively composed of single orbital A, B, and C [= sublattice interference (*34*)]. For this reason, moderate bond-order fluctuations (λ_bond_ ≳ 0.9) can induce nodeless s-wave SC gap state against spin fluctuations.

## DISCUSSION

### Importance of paramagnon interference

We have studied the exotic DW and beyond-Migdal unconventional superconductivity in kagome metal AV_3_Sb_5_ (A = K, Rb, Cs) by focusing on the paramagnon interference mechanism. This beyond-mean-field mechanism provides sizable “intersublattice” scattering, and therefore, the smectic bond order is realized in the presence of experimentally observed weak spin fluctuations.

The bond-order fluctuations naturally mediate strong pairing glue that leads to the s-wave state, consistently with recent several experiments (*20*, *21*, *29*, *38*). Thus, the origins of the star of David order, the exotic superconductivity, and the strong interplay among them are uniquely explained on the basis of the paramagnon interference mechanism. This mechanism has been overlooked previously. This previously unknown mechanism overcomes the difficulty of the sublattice interference (*34*), which leads to tiny intersite interaction in weak-coupling theories and gives rise to rich phase transitions in kagome metals. These key findings would promote future experiments on not only kagome metals but also other frustrated metals.

A great merit of the present theory is that the bond order is robustly obtained for a wide range of model parameters, as long as the band structure near the three vHS points is correctly reproduced. *U* is the only model parameter in the present theory. To clarify this merit, we make the comparison between the DW equation theory and mean-field theory.

In the mean-field theory, the instability of the charge bond order is always secondary even if large nearest-neighbor Coulomb interaction *V* is introduced. In contrast, in the DW equation theory, the charge bond-order solution is robustly obtained even when *V* = 0. This is a great merit of the present DW equation analysis. This merit remains even if both charge- and spin-channel VCs are taken into account as explained in section SF.

We also discuss interesting similarities between kagome metal and other strongly correlated metals. The paramagnon interference mechanism has been successfully applied to explain the nematic and smectic orders in Fe-based and cuprate superconductors (*4*, *6*). However, they appear only in the vicinity of the magnetic criticality, except for FeSe systems (*9*, *10*). In contrast, the smectic bond order in kagome metal appears irrespective of small spin fluctuations (α*_S_* ∼ 0.75), because of the strong geometrical frustration inherent in kagome metals. The present study would be useful to understand the recently discovered “smectic order and adjacent high-*T*_c_ state” in FeSe/SrTiO_3_ (*56*).

### Impurity effect on superconductivity

The impurity effect is one of the most notable experiments to distinguish the symmetry of the SC gap function. However, experimental reports of the impurity effect on AV_3_Sb_5_ and its theoretical analysis have been limited so far. Here, we study the nonmagnetic impurity effect on both s-wave and p-wave SC states predicted in the present theory in Fig. 4. We treat the dilute V-site impurities based on the T-matrix approximation. The impurity potential on the A site is ${\left({\hat{I}}_{\text{imp}}^{A}\right)}_{ll^{\prime }}=\delta_{l,l^{\prime }}$, where *l*, *l*′ = *A*, *A*′. In this case, the T matrix on A site is given by ${\hat{T}}^{A}={\hat{I}}_{\text{imp}}^{A}{\left(\hat{1}-{\hat{g}}^{A}{\hat{I}}_{\text{imp}}^{A}\right)}^{-1}$, where ${\hat{g}}^{A}$ is the 2 × 2 local Green function on A site. In this case, the normal self-energy is given by ${\hat{\Sigma }}^{n}=n_{\text{imp}}({\hat{T}}^{A}+{\hat{T}}^{B}+{\hat{T}}^{C})$, where *n*_imp_ is the impurity concentration. The anomalous self-energy is also given by the T matrix. Here, we consider the unitary limit case (*I*_imp_ = ∞). More detailed explanation is written in (*57*).

**Fig. 5.**
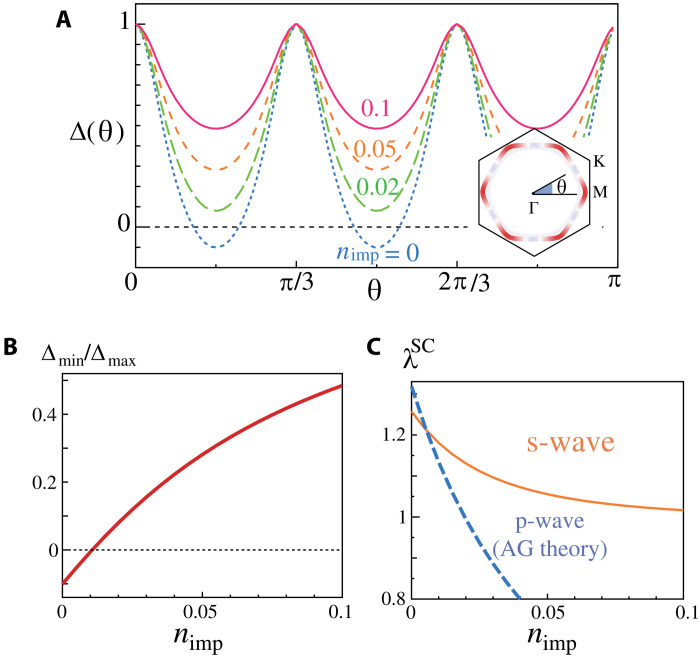
Impurity effect on superconductivity. (**A**) Obtained nodal s-wave gap function at *n*_imp_ = 0 − 0.1. (**B**) *n*_imp_ dependence of Δ_min_/Δ_max_ in the s-wave state. (**C**) *n*_imp_ dependence of the eigenvalue of s-wave and p-wave SC states. s-wave superconductivity is robust against the impurity effect, while the p-wave one is quite weak. (Here, p-wave pairing interaction is magnified by 2.7.)

Figure 5A shows the changes of the nodal s-wave SC gap function due to the impurity effect at α*_S_* = 0.75. The gap function at *n*_imp_ = 0 is the same as Fig. 4C. The accidental nodes at *n*_imp_ = 0 are lifted up due to the impurities, and the nodeless s-wave gap emerges at just *n*_imp_ = 0.02. The ratio of the minimum gap over the maximum one quickly increases with *n*_imp_ as plotted in Fig. 5B. Figure 5C shows the eigenvalues of the s-wave ($\lambda_{s}^{\text{SC}}$) and p-wave SC states ($\lambda_{p}^{\text{SC}}$). Note that $\lambda_{s(p)}^{\text{SC}}$ is proportional to s(p)-wave *T*_c_. (Here, the pairing interaction for the p-wave SC is magnified by 2.7 to make both $\lambda_{s}^{\text{SC}}$ and $\lambda_{p}^{\text{SC}}$ comparable.) $\lambda_{p}^{\text{SC}}$ drastically decreases with *n*_imp_ by following the Abrikosov-Gorkov theory. In contrast, the reduction in $\lambda_{s}^{\text{SC}}$ is much slower, and its suppression saturates when the gap becomes nearly isotropic for *n*_imp_ ≳ 0.05.

The obtained impurity-induced drastic change in the gap anisotropy is a hallmark of the s-wave SC mediated by the bond-order fluctuations. Thus, measurements of the impurity effects will be very promising toward the whole understanding of the SC phase. Note that when the p-wave SC state appears at *n*_imp_ = 0, the transition from p-wave to s-wave state is caused by introducing dilute impurities.

### *P-T* phase diagram

We discuss the *P-T* phase diagram of CsV_3_Sb_5_ in which the SC phase shows the highest *T*_c_ ∼ 8 K at the critical pressure *P*_*c*2_ ∼ 2 GPa (*T*_DW_ = 0) (*15*). Inside the bond-order phase, the second highest SC dome with *T*_c_ ∼ 6 K emerges at *P*_*c*1_ ∼ 0.7 GPa. Between *P*_*c*1_ and *P*_*c*2_, both *T*_c_ and the SC volume fraction are reduced, while the residual resistivity increases. As discussed in (*15*), these states remind us of the inhomogeneous “nearly-commensurate CDW (NCCDW)” in 1T-TaS_2_, which is realized when the correlation-driven incommensurate DW order at the FS nesting vector (*48*) is partially locked to the lattice via the electron-phonon interaction. When such an inhomogeneous DW state appears, *T*_c_ of the strongly anisotropic SC gap state should be suppressed, so the double-dome SC structure is realized.

**Fig. 6.**
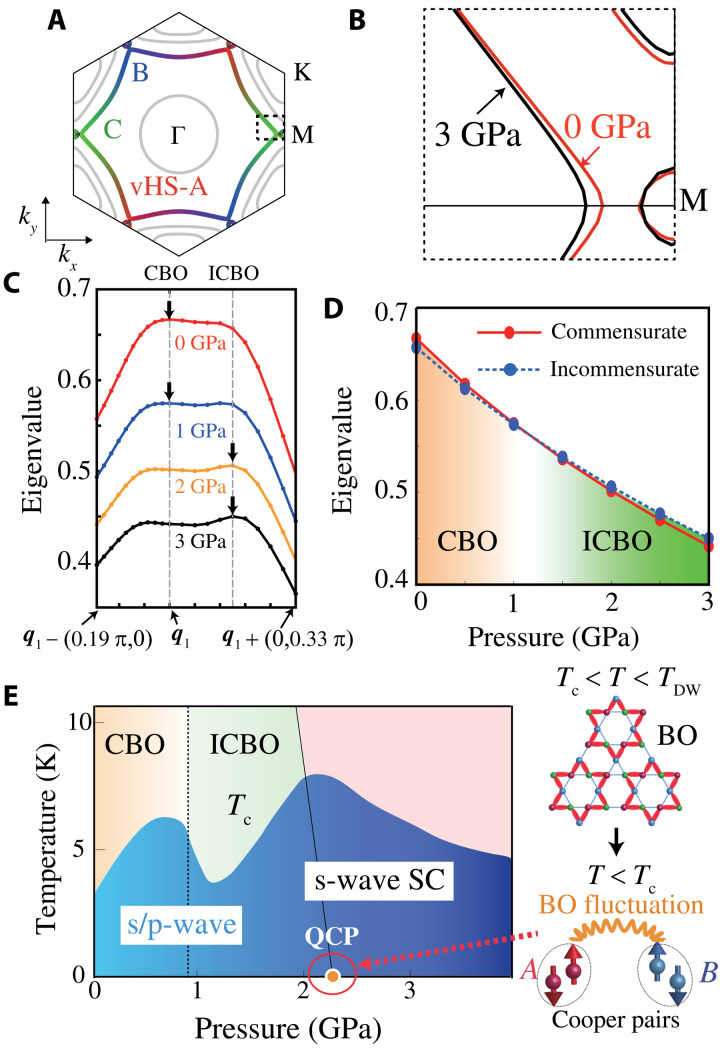
Pressure-induced C-IC bond-order transition. (**A**) FSs in the realistic 30-orbital model at ***P*** = 0. The *b*_3*g*_-orbital weight on A (red), B (blue), and C (green) sublattices is shown. (**B**) FSs around vHS points at *P* = 0 and 3 GPa. (**C**) Obtained ***q*** dependence of the eigenvalue for bond order at 0 to 3 GPa. Here, CBO (ICBO) means the commensurate (incommensurate) bond order. (**D**) Pressure dependence of the eigenvalue of the bond order. The C-IC transition emerges around *P* ∼ 1 GPa. (**E**) Schematic *P-T* phase diagram derived from the present theory.

To support this NCCDW scenario for *P* > *P*_*c*1_ (*15*), we construct realistic tight-binding models at 0 to 3 GPa based on the first-principles study, which are constructed by using Wien2k and Wannier90 software (*58*). Figure 6A shows the FSs at *P* = 0: The *b*_3*g*_-orbital FS is essentially similar to that in Fig. 1D. The *b*_3*g*_-FS at 3 GPa becomes smaller due to the pressure-induced self-doping on *b*_3*g*_-FS (∼1.5%), deviating from the vHS points as illustrated in Fig. 6B. (The change in *k*_F_ on the *k_x_* axis is Δ*k*_F_ = −0.02π.) The obtained change is reliable because it is derived from the first-principles “pressure Hamiltonian $\Delta H_{0}^{\text{DFT}}(P)$” given in section SG. The present discovered *P* dependence in the FS and its nesting vector would cause the C-IC bond-order transition.

On the basis of the derived realistic models, we perform the DW equation analysis. Figure 6C shows the obtained *q*-dependent eigenvalue, λ*_q_*, at 0 to 3 GPa with *T* = 0.04 [eV] and *U* = 2.7 [eV]. At *P* = 0, we obtain the commensurate bond-order (CBO) solution at ***q*** = ***q***_1_, so the robustness of the bond-order solution in Fig. 2 is confirmed. On the other hand, λ_*q*_1__ is quickly suppressed under pressure (over 30% at 3 GPa). Since *T*_DW_ ∝ λ_*q*_1__ qualitatively, this result is consistent with the strong suppression of the bond order under pressure in kagome metals. In the interference mechanism, the small reduction in α*_S_* induced by the pressure (just ∼0.03 at 3 GPa) causes a sizable suppression of λ_*q*_1__, as we can see in Fig. 2B. The CBO at *P* = 0 turns out to be an incommensurate one at ***q*** = ***q***_**1**_ + (0, δ) when *P* ≳ 1 GPa, by reflecting the change in the nesting condition. The *P* dependence of the bond-order eigenvalues is summarized in Fig. 6D.

In section SB, we examine the filling dependence of the bond-order solution in the present six-orbital kagome lattice model. As shown in fig. S3 (B), the C-IC bond-order transition occurs at *n* = *n*_0_ ≡ 3.82. For *n* > *n*_0_, the incommensurate bond order (ICBO) is realized due to the change in the Fermi momentum Δ*k*_F_. Thus, the C-IC transition can also be understood in the present simple six orbital model. The present theory supports the NCCDW scenario discussed in (*15*).

Next, we propose a possible scenario for the double-dome SC phase on AV_3_Sb_5_. The phase diagram based on the present scenario is schematically represented in Fig. 6E. The present bond-order fluctuation-mediated s-wave state should exhibit the highest *T*_c_ around the critical pressure *P* = *P*_*c*2_. Thus, the *T*_c_ monotonically decreases as ∣*P* − *P*_*c*2_∣ increases. In addition, the NCCDW-like inhomogeneous states triggered by the ICBO formation lead to the dip structure in *T*_c_ for *P* ≳ *P*_*c*1_. Therefore, the double-dome SC phase is naturally explained in terms of the C-IC bond-order transition.

In the other SC dome for *P* < *P*_*c*1_, both p- and s-wave SC can emerge because bond-order and spin fluctuations would be comparable. If the p-wave SC state is realized at *n*_imp_ = 0, the p-wave to s-wave SC transition will occur at *n*_imp_ ∼ 0.01 as understood in Fig. 5C.

### Future problems

In kagome metals, the bond-order state is the platform of various exotic phenomena. In this respect, the mechanism of the bond-order state should be clarified in kagome metal. The discovered quantum interference process in the present study triggers the bond-order formation, and this process would be important even below *T*_DW_. Thus, the present study paved the way for understanding the whole phase diagram.

A central open problem in the bond-order state is the time-reversal symmetry-breaking (TRSB) state. In AV_3_Sb_5_, the TRSB state has been reported by STM, Kerr effect, and Muon Spin Relaxation (μSR) measurements in (*31*, *59*, *60*). The *T*_TRSB_ ∼ 70 K is suggested by the μSR study, while *T*_TRSB_ = *T*_DW_ = 94 K is reported by the Kerr effect (*31*, *60*). The leading candidate for the TRSB is the charge LC order that accompanies the local magnetic field, as studied in cuprates for a long time (*61*, *62*).

However, the microscopic mechanism of the LC order has been unsolved. For example, the LC phase does not appear in the *U-V* phase diagram in the mean-field approximation in fig. S7. Thus, beyond-mean-field analysis is required to solve this open issue. An important clue is given by the spin-fluctuation–driven LC mechanism in frustrated metals in (*63*, *64*). This beyond-mean-field LC mechanism is general because the LC is caused by various spin/charge-channel fluctuations. Thus, new spin/charge-channel fluctuations due to the FS reconstruction below *T*_DW_ may induce the LC order in the bond-order state. Therefore, the present bond-order theory provides a starting point to understand the phase diagram of AV_3_Sb_5_.

The coexistence of the LC and the bond order is predicted based on GL theory in (*28*). The relation *T*_TRSB_ ∼ *T*_DW_ is realized when the third-order term in the GL free energy, inherent in kagome metals, is sizable. In the future, it is useful to solve the “full DW equation without linearization” in which effect of the third-order GL term is included.

Another important issue is the anomalous transport phenomena below *T*_DW_. For instance, a giant anomalous Hall effect (*65*, *66*) is observed in several kagome metals. In addition, sizable thermoelectric power and Nernst effect are reported (*67*). These transport coefficients can be calculated on the basis of the realistic tight-binding models in Fig. 6, in the presence of the bond order and the LC order. The VCs for the current due to spin/charge fluctuations would play notable roles (*68*). It is a useful future problem to study the effect of the three-dimensionality on the electronic states in kagome metals.

## MATERIALS AND METHODS

### Derivation of DW equation

Here, we derive the kernel function in the DW equation, $I_{q}^{ll^{\prime },mm^{\prime }}(k,k^{\prime })$, studied in Results. It is given as δ^2^Φ_LW_/δ*G*_*l*^′^*l*_(*k*)δ*G*_*mm*^′^_(*p*) at ***q*** = 0 in the conserving approximation scheme (*44*, *55*), where Φ_LW_ is the Luttinger-Ward function. Here, we apply the one-loop approximation for Φ_LW_ (*6*, *44*). Then, $I_{q}^{L,M}$ in this kagome model is given as

$$\begin{array}{c} I_{q}^{ll^{\prime },mm^{\prime }}(k,k^{\prime })=\sum_{b=s,c}\frac{a^{b}}{2}[-V_{\mathrm{lm},l^{\prime }m^{\prime }}^{b}(k-k^{\prime })\\ +\frac{T}{N}\sum_{p}\sum_{l_{1}l_{2},m_{1}m_{2}}V_{{\mathrm{ll}}_{1},{\mathrm{mm}}_{1}}^{b}(p+q)V_{m^{\prime }m_{2},l^{\prime }l_{2}}^{b}(p)\\ \;\times G_{l_{1}l_{2}}(k-p)G_{m_{2}m_{1}}(k^{\prime }-p)\\ +\frac{T}{N}\sum_{p}\sum_{l_{1}l_{2},m_{1}m_{2}}V_{{\mathrm{ll}}_{1},m_{2}m^{\prime }}^{b}(p+q)V_{m_{1}m,l^{\prime }l_{2}}^{b}(p)\\ \times G_{l_{1}l_{2}}(k-p)G_{m_{2}m_{1}}(k^{\prime }+p+q)] \end{array}$$ (6)

where *a*^*s*(*c*)^ = 3(1) and *p* = (***p***, *ω_l_*). ${\hat{V}}^{b}$ is the *b*-channel interaction given by ${\hat{V}}^{b}={\hat{U}}^{b}+{\hat{U}}^{b}{\hat{\chi }}^{b}{\hat{U}}^{b}$. ${\hat{U}}^{b}$ is the matrix expression of the bare multiorbital Coulomb interaction for channel *b*.

Under the uniform (***q*** = 0) DW state, the one-loop Φ_LW_ is given as $\Phi_{\text{LW}}=T\sum_{p}\;[\frac{3}{2}\text{Tr ln}\;(\hat{1}-{\hat{U}}^{s}{\hat{\chi }}^{0}(p))+\frac{1}{2}\text{Tr ln}\;(\hat{1}-{\hat{U}}^{c}{\hat{\chi }}^{0}(p))]$ with the correction terms up to *O*(*U*^2^). When the wave vector *q* of the DW state is nonzero, ${\hat{\chi }}^{0}(p)$ is replaced with ${\hat{\chi }}^{0}(p;q)$.

**Fig. 7.**
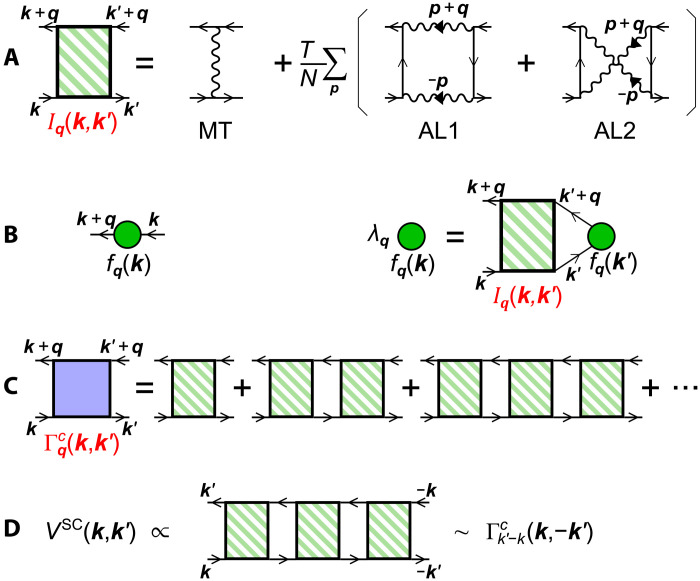
Derivations of DW equation and beyond-Migdal pairing interaction. (**A**) Charge-channel kernel function $I_{q}^{ll^{\prime },mm^{\prime }}(k,k^{\prime })$. (**B**) Linearized DW equation. (**C**) Charge-channel full four-point vertex $\Gamma_{q}^{c}(k,k^{\prime })$ obtained by solving the DW equation. (**D**) Pairing interaction due to $\Gamma_{q}^{c}$: $V^{\text{SC}}(k,k^{\prime })\propto \Gamma_{k^{\prime }-k}^{c}(k,-k^{\prime })$.

The first term of Eq. 6 corresponds to the single-magnon exchange Maki-Thompson term, and the second and third terms give two double-magnon interference AL terms. They are expressed in Fig. 7A.

The DW instability driven by nonlocal beyond-mean-field correlation ${\hat{I}}_{q}(k,k^{\prime })$ is obtained by solving the DW equation introduced in (*10*, *44*, *47*)

$$\lambda_{q}f_{q}^{ll^{\prime }}(k)=\frac{T}{N}\sum_{k^{\prime },m,m^{\prime }}K_{q}^{ll^{\prime },mm^{\prime }}(k,k^{\prime })f_{q}^{mm^{\prime }}(k^{\prime })$$ (7)

Kqll′,mm′(k,k′)=−∑m″,m‴Iqll′,m″,m‴(k,k′)×Gm″m(k′+q)Gm′m‴(k′) (8)

which is depicted in Fig. 7B. Here, λ_***q***_ is the eigenvalue that reaches unity at the transition temperature. ${\hat{f}}_{q}$ is the form factor of the DW order, which corresponds to the “symmetry-breaking in the self-energy”. By solving Eq. 7, we can obtain the optimized momentum and orbital dependences of $\hat{f}$. This mechanism has been successfully applied to explain the electronic nematic orders in Fe-based (*6*, *9*, *10*) and cuprate superconductors (*4*) and multipole orders in *f*-electron systems (*49*).

An arbitrary phase factor *e^iα^* can be multiplied to the solution of the linearized DW equation ${\hat{f}}_{q}(k)$. However, the phase factor should be determined uniquely so that ${\tilde{f}}_{q}(k)=({\hat{f}}_{q}(k,\pi T)+{\hat{f}}_{q}(k,-\pi T))/2$ satisfies the Hermite condition ${\tilde{f}}_{q}^{\mathrm{lm}}(k)={\left[{\tilde{f}}_{-q}^{\mathrm{ml}}(k+q)\right]}^{*}$.

Last, we discuss the effective interaction driven by the bond-order fluctuations. By solving the DW equation (Eq. 7), we obtain the full four-point vertex function $\Gamma_{q}^{c}(k,k^{\prime })$ that is composed of $I_{q}^{c}$ and *G*(*k* + ***q***)*G*(*k*) shown in Fig. 7C, which increases in proportion to (1 − *λ_q_*)^−1^. Thus, we obtain the relation $\Gamma_{q}^{c}(k,k^{\prime })\approx f_{q}(k){\left\{f_{q}(k^{\prime })\right\}}^{*}{\bar{I}}_{q}^{c}{\left(1-\lambda_{q}\right)}^{-1}$, which is well satisfied when λ*_q_* is close to unity.

As we will discuss in section SE, the pairing interaction due to the bond-order fluctuations is given by the full four-point vertex: $V^{\text{SC}}(k,k^{\prime })∼\Gamma_{k^{\prime }-k}^{c}(k,-k^{\prime })∼f_{k^{\prime }-k}(k){\left\{f_{k^{\prime }-k}(-k^{\prime })\right\}}^{*}{\left(1-\lambda_{q}\right)}^{-1}$, which is depicted in Fig. 7D.

Both the DW equation and the fRG method explain the nematic and smectic bond order in single-orbital square lattice Hubbard models (*46*, *47*) and anisotropic triangular lattice ones (*4*). This fact means that higher-order diagrams other than MT or AL terms, which are included in the fRG method, are not essential in explaining the bond order. Note that the contributions away from the conduction bands are included into N-patch fRG by applying the RG + cRPA method (*4*, *4*, *46*).

## Supplementary Materials

### This PDF file includes:

Model Hamiltonian and RPA Robustness of bond-order solution in the DW equation Unfolded Fermi surface under triple-q state GL free energy in D6h kagome model Derivation of SC gap equation Comparison between the present DW equation theory and mean-field theory Realistic model Hamiltonian based on the first-principles study Figs. S1 to S9

## References

[1] E. Fradkin, S. A. Kivelson, Ineluctable complexity. Nat. Phys. 8, 864–866 (2012).

[2] J. C. S. Davis, D.-H. Lee, Concepts relating magnetic interactions, intertwined electronic orders, and strongly correlated superconductivity. Proc. Natl. Acad. Sci. U.S.A. 110, 17623–17630 (2013). 24114268 10.1073/pnas.1316512110 PMC3816467

[3] T. Shibauchi, T. Hanaguri, Y. Matsuda, Exotic superconducting states in FeSe-based materials. J. Phys. Soc. Jpn. 89, 102002 (2020).

[4] R. Tazai, Y. Yamakawa, M. Tsuchiizu, H. Kontani, *d*- and *p*-wave quantum liquid crystal orders in cuprate superconductors, κ-(BEDT-TTF)_2_X, and coupled chain hubbard models: Functional renormalization group analysis. J. Phys. Soc. Jpn. 90, 111012 (2021).

[5] H. Kontani, T. Saito, S. Onari, Origin of orthorhombic transition, magnetic transition, and shear-modulus softening in iron pnictide superconductors: Analysis based on the orbital fluctuations theory. Phys. Rev. B 84, 024528 (2011).

[6] S. Onari, H. Kontani, Self-consistent vertex correction analysis for iron-based superconductors: Mechanism of coulomb interaction-driven orbital fluctuations. Phys. Rev. Lett. 109, 137001 (2012). 23030111 10.1103/PhysRevLett.109.137001

[7] M. Tsuchiizu, Y. Ohno, S. Onari, H. Kontani, Orbital nematic instability in the two-orbital hubbard model: Renormalization-group + constrained RPA analysis. Phys. Rev. Lett. 111, 057003 (2013). 23952433 10.1103/PhysRevLett.111.057003

[8] Y. Yamakawa, H. Kontani, Spin-fluctuation-driven nematic charge-density wave in cuprate superconductors: Impact of Aslamazov-Larkin vertex corrections. Phys. Rev. Lett. 114, 257001 (2015). 26197139 10.1103/PhysRevLett.114.257001

[9] Y. Yamakawa, S. Onari, H. Kontani, Nematicity and magnetism in FeSe and other families of Fe-based superconductors. Phys. Rev. X 6, 021032 (2016).

[10] S. Onari, Y. Yamakawa, H. Kontani, Sign-reversing orbital polarization in the nematic phase of fese due to the *C*_2_ symmetry breaking in the self-energy. Phys. Rev. Lett. 116, 227001 (2016). 27314734 10.1103/PhysRevLett.116.227001

[11] A. V. Chubukov, M. Khodas, R. M. Fernandes, Magnetism, superconductivity, and spontaneous orbital order in iron-based superconductors: Which comes first and why? Phys. Rev. X 6, 041045 (2016).

[12] R. M. Fernandes, P. P. Orth, J. Schmalian, Intertwined vestigial order in quantum materials: Nematicity and beyond. Annu. Rev. Condens. Matter Phys. 10, 133–154 (2019).

[13] B. R. Ortiz, L. C. Gomes, J. R. Morey, M. Winiarski, M. Bordelon, J. S. Mangum, I. W. H. Oswald, J. A. Rodriguez-Rivera, J. R. Neilson, S. D. Wilson, E. Ertekin, T. M. McQueen, E. S. Toberer, New kagome prototype materials: Discovery of KV_3_Sb_5_, RbV_3_Sb_5_, and CsV_3_Sb_5_. Phys. Rev. Mater. 3, 094407 (2019).

[14] B. R. Ortiz, S. M. L. Teicher, Y. Hu, J. L. Zuo, P. M. Sarte, E. C. Schueller, A. M. M. Abeykoon, M. J. Krogstad, S. Rosenkranz, R. Osborn, R. Seshadri, L. Balents, J. He, S. D. Wilson, CsV_3_Sb_5_: A ℤ_2_ topological kagome metal with a superconducting ground state. Phys. Rev. Lett. 125, 247002 (2020). 33412053 10.1103/PhysRevLett.125.247002

[15] F. H. Yu, D. H. Ma, W. Z. Zhuo, S. Q. Liu, X. K. Wen, B. Lei, J. J. Ying, X. H. Chen, Unusual competition of superconductivity and charge-density-wave state in a compressed topological kagome metal. Nat. Commun. 12, 3645 (2021). 34112779 10.1038/s41467-021-23928-w PMC8192749

[16] K. Y. Chen, N. N. Wang, Q. W. Yin, Y. H. Gu, K. Jiang, Z. J. Tu, C. S. Gong, Y. Uwatoko, J. P. Sun, H. C. Lei, J. P. Hu, J.-G. Cheng, Double superconducting dome and triple enhancement of *T_c_* in the kagome superconductor CsV_3_Sb_5_ under high pressure. Phys. Rev. Lett. 126, 247001 (2021). 34213920 10.1103/PhysRevLett.126.247001

[17] B. R. Ortiz, P. M. Sarte, E. M. Kenney, M. J. Graf, S. M. L. Teicher, R. Seshadri, S. D. Wilson, Superconductivity in the ℤ_2_ kagome metal KV_3_Sb_5_. Phys. Rev. Mater. 5, 034801 (2021).

[18] Q. Yin, Z. Tu, C. Gong, Y. Fu, S. Yan, H. Lei, Superconductivity and normal-state properties of kagome metal RbV−3*Sb*_5_ single crystals. Chin. Phys. Lett. 38, 037403 (2021).

[19] C. C. Zhao, L. S. Wang, W. Xia, Q. W. Yin, J. M. Ni, Y. Y. Huang, C. P. Tu, Z. C. Tao, Z. J. Tu, C. S. Gong, H. C. Lei, Y. F. Guo, X. F. Yang, S. Y. Li, Nodal superconductivity and superconducting domes in the topological Kagome metal CsV3Sb5. arXiv:2102.08356 (2021).

[20] W. Duan, Z. Nie, S. Luo, F. Yu, B. R. Ortiz, L. Yin, H. Su, F. Du, A. Wang, Y. Chen, X. Lu, J. Ying, S. D. Wilson, X. Chen, Y. Song, H. Yuan, Nodeless superconductivity in the kagome metal CsV3Sb5. Sci. China Phys. Mech. Astron. 64, 107462 (2021).

[21] R. Gupta, D. Das, C. H. Mielke III, Z. Guguchia, T. Shiroka, C. Baines, M. Bartkowiak, H. Luetkens, R. Khasanov, Q. Yin, Z. Tu, C. Gong, H. Lei, Microscopic evidence for anisotropic multigap superconductivity in the CsV−3*Sb*_5_ kagome superconductor. arXiv:2108.01574 (2021).

[22] R. Lou, A. Fedorov, Q. Yin, A. Kuibarov, Z. Tu, C. Gong, E. F. Schwier, B. Büchner, H. Lei, S. Borisenko, Charge-density-wave-induced peak-dip-hump structure and flat band in the kagome superconductor CsV_3_*Sb*_5_. arXiv:2106.06497 (2021). 10.1103/PhysRevLett.128.036402 35119899

[23] T. Park, M. Ye, L. Balents, Electronic instabilities of kagome metals: Saddle points and Landau theory. Phys. Rev. B 104, 035142 (2021).

[24] H. Tan, Y. Liu, Z. Wang, B. Yan, Charge density waves and electronic properties of superconducting kagome metals. Phys. Rev. Lett. 127, 046401 (2021). 34355948 10.1103/PhysRevLett.127.046401

[25] X. Wu, T. Schwemmer, T. Müller, A. Consiglio, G. Sangiovanni, D. Di Sante, Y. Iqbal, W. Hanke, A. P. Schnyder, M. M. Denner, M. H. Fischer, T. Neupert, R. Thomale, Nature of unconventional pairing in the kagome superconductors *A*V_3_Sb_5_ (*A* = K, Rb, Cs). Phys. Rev. Lett. 127, 177001 (2021). 34739258 10.1103/PhysRevLett.127.177001

[26] M. M. Denner, R. Thomale, T. Neupert, Analysis of charge order in the kagome metal *A*V_3_Sb_5_ (*A* = K, Rb, Cs). Phys. Rev. Lett. 127, 217601 (2021). 34860107 10.1103/PhysRevLett.127.217601

[27] C. Setty, H. Hu, L. Chen, Q. Si, Electron correlations and *T*-breaking density wave order in a ℤ_2_ kagome metal. arXiv:2105.15204 (2021).

[28] Y.-P. Lin, R. M. Nandkishore, Complex charge density waves at Van Hove singularity on hexagonal lattices: Haldane-model phase diagram and potential realization in the kagome metals *AV*_3_Sb_5_ (*A*=K, Rb, Cs). Phys. Rev. B 104, 045122 (2021).

[29] C. Mu, Q. Yin, Z. Tu, C. Gong, H. Lei, Z. Li, J. Luo, S-wave superconductivity in kagome metal CsV−3*Sb*_5_ revealed by ^121/123^Sb NQR and ^51^V NMR measurements. Chin. Phys. Lett. 38, 077402 (2021).

[30] D. W. Song, L. X. Zheng, F. H. Yu, J. Li, L. P. Nie, M. Shan, D. Zhao, S. J. Li, B. L. Kang, Z. M. Wu, Y. B. Zhou, K. L. Sun, K. Liu, X. G. Luo, Z. Y. Wang, J. J. Ying, X. G. Wan, T. Wu, X. H. Chen, Orbital ordering and fluctuations in a kagome superconductor CsV3Sb5. arXiv:2104.09173 (2021).

[31] Y.-X. Jiang, J.-X. Yin, M. M. Denner, N. Shumiya, B. R. Ortiz, G. Xu, Z. Guguchia, J. He, M. S. Hossain, X. Liu, J. Ruff, L. Kautzsch, S. S. Zhang, G. Chang, I. Belopolski, Q. Zhang, T. A. Cochran, D. Multer, M. Litskevich, Z.-J. Cheng, X. P. Yang, Z. Wang, R. Thomale, T. Neupert, S. D. Wilson, M. Z. Hasan, Unconventional chiral charge order in kagome superconductor KV3Sb5. Nat. Mater. 20, 1353–1357 (2021). 34112979 10.1038/s41563-021-01034-y

[32] H. Li, H. Zhao, B. R. Ortiz, T. Park, M. Ye, L. Balents, Z. Wang, S. D. Wilson, I. Zeljkovic, Rotation symmetry breaking in the normal state of a kagome superconductor KV3Sb5. arXiv:2104.08209 (2021).

[33] H. Li, T. T. Zhang, T. Yilmaz, Y. Y. Pai, C. E. Marvinney, A. Said, Q. W. Yin, C. S. Gong, Z. J. Tu, E. Vescovo, C. S. Nelson, R. G. Moore, S. Murakami, H. C. Lei, H. N. Lee, B. J. Lawrie, H. Miao, Observation of unconventional charge density wave without acoustic phonon anomaly in kagome superconductors *A*V_3_Sb_5_ (*A* = Rb, Cs). Phys. Rev. X 11, 031050 (2021).

[34] M. L. Kiesel, C. Platt, R. Thomale, Unconventional fermi surface instabilities in the kagome hubbard model. Phys. Rev. Lett. 110, 126405 (2013). 25166827 10.1103/PhysRevLett.110.126405

[35] W.-S. Wang, Z.-Z. Li, Y.-Y. Xiang, Q.-H. Wang, Competing electronic orders on kagome lattices at van Hove filling. Phys. Rev. B 87, 115135 (2013).

[36] J. Wen, A. Rüegg, C.-C. J. Wang, G. A. Fiete, Interaction-driven topological insulators on the kagome and the decorated honeycomb lattices. Phys. Rev. B 82, 075125 (2010).

[37] E. M. Kenney, B. R. Ortiz, C. Wang, S. D. Wilson, M. J. Graf, Absence of local moments in the kagome metal KV−3*Sb*_5_ as determined by muon spin spectroscopy. J. Phys. Condens. Matter 33, 235801 (2021). 10.1088/1361-648X/abe8f9 33621958

[38] H.-S. Xu, Y.-J. Yan, R. Yin, W. Xia, S. Fang, Z. Chen, Y. Li, W. Yang, Y. Guo, D.-L. Feng, Multiband Superconductivity with sign-preserving order parameter in kagome superconductor CsV_3_Sb_5_. Phys. Rev. Lett. 127, 187004 (2021). 34767411 10.1103/PhysRevLett.127.187004

[39] Y. Wang, S. Yang, P. K. Sivakumar, B. R. Ortiz, S. M. L. Teicher, H. Wu, A. K. Srivastava, C. Garg, D. Liu, S. S. P. Parkin, E. S. Toberer, T. McQueen, S. D. Wilson, M. N. Ali, Proximity-induced spin-triplet superconductivity and edge supercurrent in the topological kagome metal, K − 1 − xV_3_Sb_5_. arXiv:2012.05898 (2020).

[40] S. Ni, S. Ma, Y. Zhang, J. Yuan, H. Yang, Z. Lu, N. Wang, J. Sun, Z. Zhao, D. Li, S. Liu, H. Zhang, H. Chen, K. Jin, J. Cheng, L. Yu, F. Zhou, X. Dong, J. Hu, H.-J. Gao, Z. Zhao, Anisotropic superconducting properties of kagome metal CsV−3Sb_5_. Chin. Phys. Lett. 38, 057403 (2021).

[41] Y. Xiang, Q. Li, Y. Li, W. Xie, H. Yang, Z. Wang, Y. Yao, H.-H. Wen, Twofold symmetry of c-axis resistivity in topological kagome superconductor CsV_3_Sb_5_ with in-plane rotating magnetic field. Nat. Commun. 12, 6727 (2021). 34795303 10.1038/s41467-021-27084-z PMC8602318

[42] Z. Liang, X. Hou, F. Zhang, W. Ma, P. Wu, Z. Zhang, F. Yu, J.-J. Ying, K. Jiang, L. Shan, Z. Wang, X.-H. Chen, Three-dimensional charge density wave and surface-dependent vortex-core states in a kagome superconductor CsV_3_Sb_5_. Phys. Rev. X 11, 031026 (2021).

[43] R. Tazai, H. Kontani, Fully gapped *s*-wave superconductivity enhanced by magnetic criticality in heavy-fermion systems. Phys. Rev. B 98, 205107 (2018).

[44] S. Onari, H. Kontani, Hidden antiferronematic order in Fe-based superconductor BaFe_2_As_2_ and NaFeAs above *T_s_*. Phys. Rev. Res. 2, 042005(R) (2020).

[45] A. V. Chubukov, P. Wölfle, Quasiparticle interaction function in a two-dimensional Fermi liquid near an antiferromagnetic critical point. Phys. Rev. B 89, 045108 (2014).

[46] M. Tsuchiizu, K. Kawaguchi, Y. Yamakawa, H. Kontani, Multistage electronic nematic transitions in cuprate superconductors: A functional-renormalization-group analysis. Phys. Rev. B 97, 165131 (2018).

[47] K. Kawaguchi, Y. Yamakawa, M. Tsuchiizu, H. Kontani, Competing unconventional charge-density-wave states in cuprate superconductors: Spin-fluctuation-driven mechanism. J. Phys. Soc. Jpn. 86, 063707 (2017).

[48] T. Hirata, Y. Yamakawa, S. Onari, H. Kontani, Unconventional orbital charge density wave mechanism in the transition metal dichalcogenide 1*T*−TaS_2_. Phys. Rev. Res. 3, L032053 (2021).

[49] R. Tazai, H. Kontani, Multipole fluctuation theory for heavy fermion systems: Application to multipole orders in CeB_6_. Phys. Rev. B 100, 241103(R) (2019).

[50] K. Nakayama, Y. Li, T. Kato, M. Liu, Z. Wang, T. Takahashi, Y. Yao, T. Sato, Multiple energy scales and anisotropic energy gap in the charge-density-wave phase of the kagome superconductor CsV_3_Sb_5_. Phys. Rev. B 104, L161112 (2021).

[51] Z. Liu, N. Zhao, Q. Yin, C. Gong, Z. Tu, M. Li, W. Song, Z. Liu, D. Shen, Y. Huang, K. Liu, H. Lei, S. Wang, Charge-density-wave–induced bands renormalization and energy gaps in a kagome superconductor RbV_3_Sb_5_. Phys. Rev. X 11, 041010 (2021).

[52] Z. Wang, S. Ma, Y. Zhang, H. Yang, Z. Zhao, Y. Ou, Y. Zhu, S. Ni, Z. Lu, H. Chen, K. Jiang, L. Yu, Y. Zhang, X. Dong, J. Hu, H.-J. Gao, Z. Zhao, Distinctive momentum dependent charge-density-wave gap observed in CsV−3*Sb*_5_ superconductor with topological kagome lattice. arXiv:2104.05556 (2021).

[53] Y. Hu, X. Wu, B. R. Ortiz, S. Ju, X. Han, J. Z. Ma, N. C. Plumb, M. Radovic, R. Thomale, S. D. Wilson, A. P. Schnyder, M. Shi, Rich nature of van hove singularities in kagome superconductor CsV−3*Sb*_5_. arXiv:2106.05922 (2021). 10.1038/s41467-022-29828-x PMC9038924 35468883

[54] Y. Luo, S. Peng, S. M. L. Teicher, L. Huai, Y. Hu, B. R. Ortiz, Z. Wei, J. Shen, Z. Ou, B. Wang, Y. Miao, M. Guo, M. Shi, S. D. Wilson, J.-F. He, Distinct band reconstructions in kagome superconductor CsV−3*Sb*_5_. arXiv:2106.01248 (2021).

[55] G. Baym, Self-consistent approximations in many-body systems. Phys. Rev. 127, 1391–1401 (1962).

[56] Y. Yuan, X. Fan, X. Wang, K. He, Y. Zhang, Q.-K. Xue, W. Li, Incommensurate smectic phase in close proximity to the high-Tc superconductor FeSe/SrTiO_3_. Nat. Commun. 12, 2196 (2021). 33850158 10.1038/s41467-021-22516-2 PMC8044195

[57] H. Kontani, S. Onari, Orbital-fluctuation-mediated superconductivity in iron pnictides: Analysis of the five-orbital hubbard-holstein model. Phys. Rev. Lett. 104, 157001 (2010). 20482011 10.1103/PhysRevLett.104.157001

[58] A. A. Tsirlin, P. Fertey, B. R. Ortiz, B. Klis, V. Merkl, M. Dressel, S. D. Wilson, E. Uykur, Role of Sb in the superconducting kagome metal CsV−3*Sb*_5_ revealed by its anisotropic compression. arXiv:2105.01397 (2021).

[59] C. Mielke III, D. Das, J.-X. Yin, H. Liu, R. Gupta, C. N. Wang, Y.-X. Jiang, M. Medarde, X. Wu, H. C. Lei, J. J. Chang, P. Dai, Q. Si, H. Miao, R. Thomale, T. Neupert, Y. Shi, R. Khasanov, M. Z. Hasan, H. Luetkens, Z. Guguchia, Time-reversal symmetry-breaking charge order in a correlated kagome superconductor, arXiv:2106.13443 (2021). 10.1038/s41586-021-04327-z 35140387

[60] Q. Wu, Z. X. Wang, Q. M. Liu, R. S. Li, S. X. Xu, Q. W. Yin, C. S. Gong, Z. J. Tu, H. C. Lei, T. Dong, N. L. Wang, The large static and pump-probe Kerr effect with two-fold rotation symmetry in kagome metal CsV−3*Sb*_5_. arXiv:2110.11306 (2021).

[61] C. M. Varma, Non-Fermi-liquid states and pairing instability of a general model of copper oxide metals. Phys. Rev. B 55, 14554–14580 (1997).

[62] I. Affleck, J. B. Marston, Large-n limit of the Heisenberg-Hubbard model: Implications for high-*T_c_* superconductors. Phys. Rev. B 37, 3774–3777 (1988). 10.1103/physrevb.37.3774 9944997

[63] H. Kontani, Y. Yamakawa, R. Tazai, S. Onari, Odd-parity spin-loop-current order mediated by transverse spin fluctuations in cuprates and related electron systems. Phys. Rev. Res. 3, 013127 (2021).

[64] R. Tazai, Y. Yamakawa, H. Kontani, Emergence of charge loop current in the geometrically frustrated Hubbard model: A functional renormalization group study. Phys. Rev. B 103, L161112 (2021).

[65] S.-Y. Yang, Y. Wang, B. R. Ortiz, D. Liu, J. Gayles, E. Derunova, R. Gonzalez-Hernandez, L. Šmejkal, Y. Chen, S. S. P. Parkin, S. D. Wilson, E. S. Toberer, T. McQueen, M. N. Ali, Giant, unconventional anomalous Hall effect in the metallic frustrated magnet candidate, KV_3_Sb_5_. Sci. Adv. 6, eabb6003 (2020). 32789181 10.1126/sciadv.abb6003 PMC7399694

[66] F. H. Yu, T. Wu, Z. Y. Wang, B. Lei, W. Z. Zhuo, J. J. Ying, X. H. Chen, Concurrence of anomalous Hall effect and charge density wave in a superconducting topological kagome metal. Phys. Rev. B 104, L041103 (2021).

[67] Y. Gan, W. Xia, L. Zhang, K. Yang, X. Mi, A. Wang, Y. Chai, Y. Guo, X. Zhou, M. He, Magneto-Seebeck effect and ambipolar Nernst effect in the CsV_3_Sb_5_ superconductor. Phys. Rev. B 104, L180508 (2021).

[68] H. Kontani, Anomalous transport phenomena in Fermi liquids with strong magnetic fluctuations. Rep. Prog. Phys. 71, 026501 (2008).

