# Supplementary material for: Mechanism of exotic density-wave and beyond-Migdal unconventional superconductivity in kagome metal AV3Sb5 (A = K, Rb, Cs)
Source: Sci Adv. 2022 Apr 1;8(13):eabl4108. doi: 10.1126/sciadv.abl4108 (PMC10938589; doi:10.1126/sciadv.abl4108)
Supplement: Supplementary file 1 — Model Hamiltonian and RPA Robustness of bond-order solution in the DW equation Unfolded Fermi surface under triple-q state GL free energy in D6h kagome model Derivation of SC gap equation Comparison between the present DW equation theory and mean-field theory Realistic model Hamiltonian based on the first-principles study Figs. S1 to S9 [file sciadv.abl4108_sm.pdf]

Science Advances
AAAS

Supplementary Materials for

Mechanism of exotic density-wave and beyond-Migdal unconventional superconductivity in kagome metal AV_3Sb_5 (A = K, Rb, Cs)

Rina Tazai*, Youichi Yamakawa, Seiichiro Onari, Hiroshi Kontani*

*Corresponding author. Email: tazai@s.phys.nagoya-u.ac.jp (R.T.); kon@slab.phys.nagoya-u.ac.jp (H.K.)

Published 1 April 2022, *Sci. Adv.* **8**, eabl4108 (2022)
DOI: 10.1126/sciadv.abl4108

This PDF file includes:

Model Hamiltonian and RPA
Robustness of bond-order solution in the DW equation
Unfolded Fermi surface under triple-q state
GL free energy in D6h kagome model
Derivation of SC gap equation
Comparison between the present DW equation theory and mean-field theory
Realistic model Hamiltonian based on the first-principles study
Figs. S1 to S9

A: Model Hamiltonian and RPA

In the main text, we analyzed the kagome lattice model shown in Fig. 1 (b) introduced in Ref. [25]. In this model, a unit cell contains three sites (A, B, C), and each site possesses two orbitals (b_{3g} and b_{2g}). In the theoretical analysis, it is more convenient to study a completely equivalent "six-orbital triangular lattice model" in Fig. S1: It is derived from the kagome lattice model by shifting three apical sites (A, B, C) of each upper triangular to its center, without changing the hopping integrals and the Coulomb interaction terms. One of the great merits of analyzing this triangular model is that any inter-site vector $\boldsymbol{r}_i - \boldsymbol{r}_j$ is equal to a translation vector, and therefore functions in the momentum space (such as $\chi^s_{ll',mm'}(\boldsymbol{q})$ and $f^{lm}_{\boldsymbol{q}}(\boldsymbol{k})$) become periodic in the first Brillouin zone (BZ). For this reason, we perform the numerical study in the main text based on the triangular lattice model in Fig. S1.

Next, we explain the multiorbital Coulomb interaction. The matrix expression of the spin-channel Coulomb interaction is

$$U^s_{l_1l_2,l_3l_4} = \begin{cases} U, & l_1 = l_2 = l_3 = l_4 \\ U', & l_1 = l_3 \neq l_2 = l_4 \\ J, & l_1 = l_2 \neq l_3 = l_4 \\ J', & l_1 = l_4 \neq l_2 = l_3 \end{cases} \tag{S1}$$

in the case that $l_1 \sim l_4$ are orbitals (X, X') at site X (=A,B,C). In other cases, $U^s_{l_1l_2,l_3l_4} = 0$.

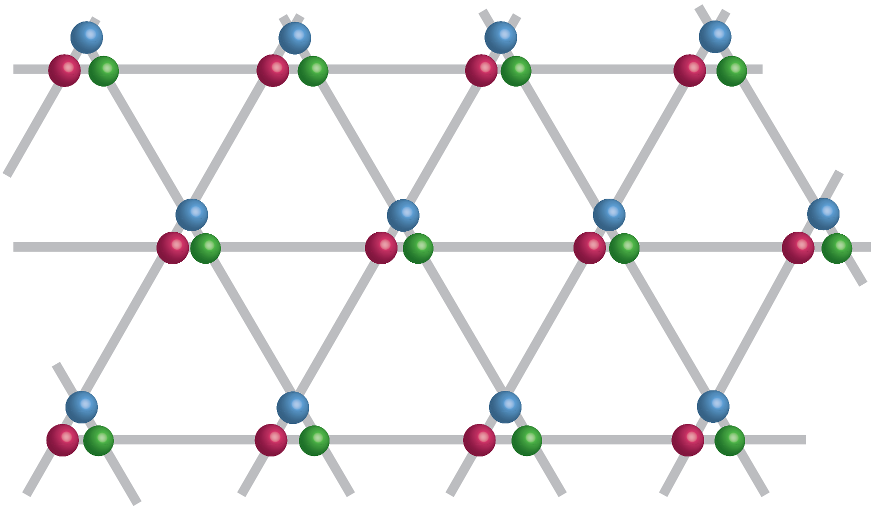

FIG. S1: **Six-orbital triangular lattice model:** In this model, three apical sites (A, B, C) of each upper triangular site in the kagome lattice are located at the same position. This model is convenient for the numerical study because both intra- and inter-orbital susceptibilities become periodic in the first BZ.

Also, the matrix expression of the charge-channel Coulomb interaction is

$$U^c_{l_1 l_2, l_3 l_4} = \begin{cases} -U, & l_1 = l_2 = l_3 = l_4 \\ U' - 2J, & l_1 = l_3 \neq l_2 = l_4 \\ -2U' + J, & l_1 = l_2 \neq l_3 = l_4 \\ -J', & l_1 = l_4 \neq l_2 = l_3 \end{cases} \tag{S2}$$

in the case that $l_1 \sim l_4$ are orbitals $(X,\ X')$ at site X (=A,B,C). In other cases, $U^c_{l_1 l_2, l_3 l_4} = 0$. Here, U (U') is the intra-orbital (inter-orbital) Coulomb interaction, J is the Hund's coupling, and J' is the pair hopping term. In the main text, we assume the relations $U = U' + 2J$ and $J = J'$, and set the constraint $J/U = 0.10$. The obtained results are not sensitive to the ratio J/U.

The spin (charge) susceptibility in the RPA, $\chi^{s(c)}_{ll',mm'}(q)$, is given by

$$\hat{\chi}^{s(c)}(q) = \hat{\chi}^0(q)(\hat{1} - \hat{U}^{s(c)}\hat{\chi}^0(q))^{-1}, \tag{S3}$$

where the element of the irreducible susceptibility is $\chi^0_{ll',mm'}(q) = -\frac{T}{N}\sum_k G_{lm}(k+q)G_{m'l'}(k)$. $G_{lm}(k)$ is the (l,m) element of the electron Green function: $\hat{G} = (\epsilon_n \hat{1} - \hat{H}_0(\boldsymbol{k}))^{-1}$.

In the present model, $\chi^s_{ll',mm'}(q)$ is small unless all orbitals belong to b_{3g}. Also, $\chi^s_{ll',mm'}(q)$

becomes large only when $l = l' = m = m'$ and $l = A$ or B or C. The spin susceptibility in the present model is shown in Fig. 1 (e) in the main text.

B: Robustness of bond-order solution in the DW equation

In the main text, we presented the numerical results for $n = 3.8$. In this case, the b_{3g}-orbital FS is very close to the vHS points on the BZ boundary as shown in Fig.1 (d), consistently with recent ARPES reports. In this model, the van Hove filling is $n_{\rm vHS} = 3.71$, and the single large FS around Γ point is divided into two pockets around K and K' points for $n < n_{\rm vHS}$. Thus, it is important to verify the robustness of numerical results for different electron filling n.

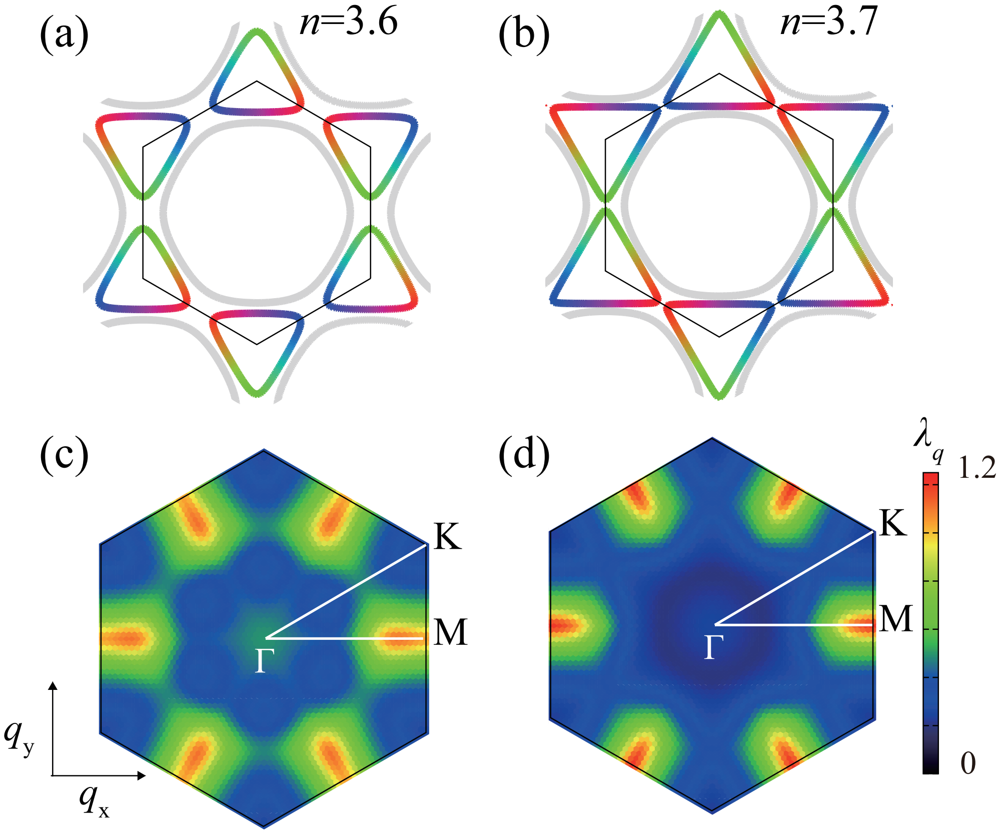

FIG. S2: **Robustness of bond-order solution:** (a) FS for $n = 3.6$ and (b) FS for $n = 3.7$. (c) $\boldsymbol{q}$-dependence of the eigenvalue for $n = 3.6$ (incommensurate) and (d) that for $n = 3.7$ (commensurate). Here, $U = 1.34$ (1.25) for $n = 3.6$ (3.7). The unit of energy (in Coulomb interaction, hopping integral, and temperature) is eV.

First, we study the case of $n < 3.8$. Figures S2 (a) and (b) represent the FS at $n = 3.6$ and 3.7, respectively. The obtained eigenvalue of the DW equation at $n = 3.6$ and 3.7 in the case of $\alpha_S = 0.8$ is shown in Figs. S2 (c) and (d), respectively. Thus, the commensurate-incommensurate (C-IC) transition occurs between $n = 3.7$ and 3.6. In both cases, the smectic bond-order at $\boldsymbol{q} \approx \boldsymbol{q}_n$ $(n = 1, 2, 3)$ is satisfactorily obtained, irrespective of the Lifshitz transition at $n_{\rm vHS} = 3.71$. Therefore, we conclude that the strong electron correlation due to the three vHS points is essential for the formation of the bond-order, while the shape and the topology of FS are not essential. We stress that the second-largest eigenvalue is considerably smaller than the present bond-order eigenvalue at $\boldsymbol{q} = \boldsymbol{q}_1$.

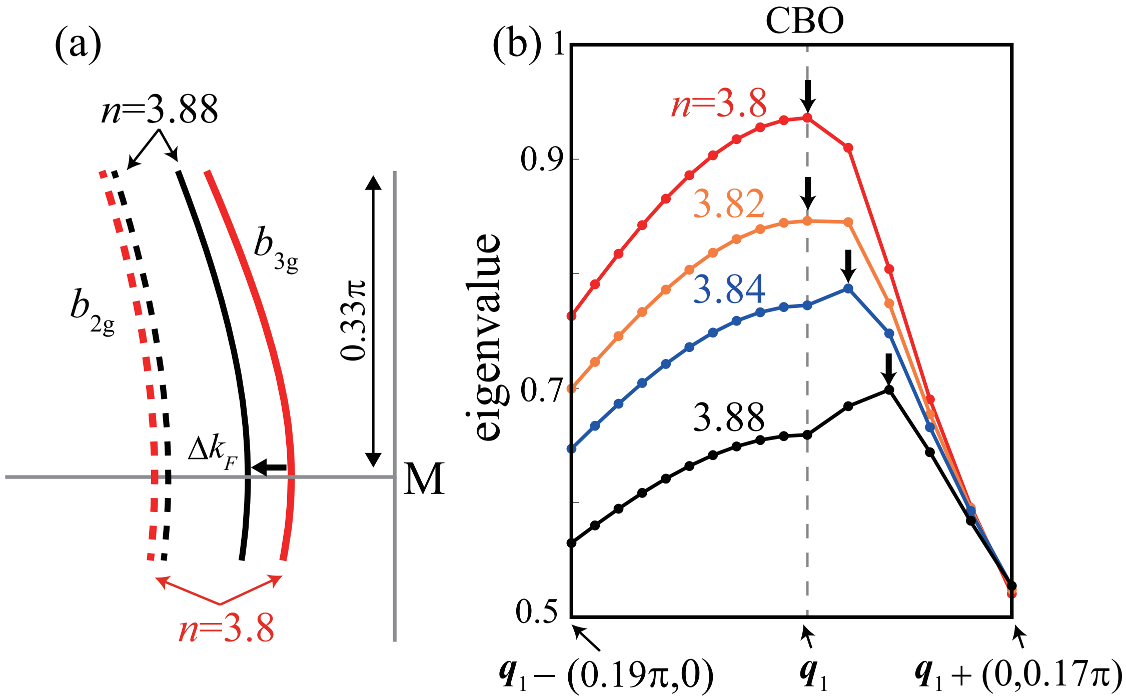

FIG. S3: **C-IC bond-order transition in the six orbital model:** (a) n-dependence of the FS in the six orbital model around M point. (The whole FS at $n = 3.8$ is shown in Fig. 1 (a).) The reduction in the b_{3g}-orbital Fermi momentum is expressed as $\Delta k_{\rm F}$. (b) Obtained $\boldsymbol{q}$-dependence of the eigenvalue for bond-order at $T = 0.01$ and $\alpha_S = 0.8$. Here, CBO means the commensurate bond-order. The C-IC transition occurs at $n = n_0 \equiv 3.82$. The wavevector of the DW state is $\boldsymbol{q}_{DW} = \boldsymbol{q}_1 + (0, \delta)$, and $\delta > 0$ for $n > n_0$. Here, $U = 1.18$ (1.21) at $n = 3.80$ (3.88).

Next, we study the case of $n \geq 3.8$. Figures S3 (a) and (b) exhibits the FSs and the $\boldsymbol{q}$-dependence of the bond-order eigenvalue $\lambda_{\boldsymbol{q}}$, respectively, in the present simple six orbital

model at $T = 0.01$. U is set to satisfy $\alpha_S = 0.80$ at each n. For $n \le n_0 \equiv 3.82$, the wavevector of the bond-order is commensurate: $\boldsymbol{q}_{\rm DW} = \boldsymbol{q}_1 \equiv (\frac{2}{\sqrt{3}}\pi, 0)$. For $n > n_0$, it changes to incommensurate at $\boldsymbol{q}_{\rm DW} = \boldsymbol{q}_1 + (0, \delta)$. The realized electron-doping in the b_{3g}-orbital FS is $\Delta n_{b_{3g}} = 0.7(n - n_0)$. The induced shift of the b_{3g}-orbital Fermi momentum on the k_x-axis is $\Delta k_{\rm F} = -0.62\pi \times \Delta n_{b_{3g}}$. Thus, $\Delta k_{\rm F} = -0.02\pi$ is realized when $\Delta n_{b_{3g}} = 0.033$ (or $n = n_0 + 0.046$). On the other hand, in the realistic 30 orbital model in SM G, $\Delta k_{\rm F} = -0.02\pi$ is induced by the self-doping ($\sim 1.5\%$) at $P = 3$GPa. Thus, the C-IC bond-order transition can also be understood based on the present simple six orbital Hubbard model.

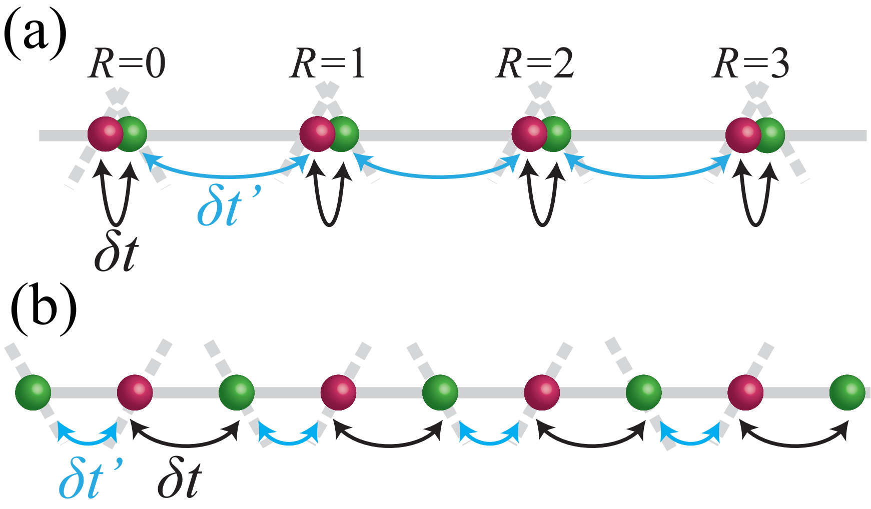

FIG. S4: **Bond-order solution in kagome metal:** (a) Nearest-neighbor hopping modulations δt and $\delta t'$ under the bond-order at $\boldsymbol{q} = \boldsymbol{q}_3$ in the triangular lattice model in Fig. S1. (b) δt and $\delta t'$ in the kagome lattice model in Fig. 1 (b).

These numerical results indicate the importance of the vHS points. Hereafter, we discuss a simplified DW equation by focusing on the vHS points in order to understand why bond-order is obtained. For the bond-order at $\boldsymbol{q} = \boldsymbol{q}_3$, only vHS points A and C are essential, so we consider a simple two-component form factor $(f_1, f_2) \equiv (f^{CA}_{\boldsymbol{q}_3}(\boldsymbol{k}_{\rm A}), f^{AC}_{\boldsymbol{q}_3}(\boldsymbol{k}_{\rm C}))$. Then, the DW equation at $\boldsymbol{q} = \boldsymbol{q}_3$ is given as

$$\lambda \begin{pmatrix} f_1 \\ f_2 \end{pmatrix} \sim N(0) \begin{pmatrix} g_{\rm back} & g_{\rm um} \\ g_{\rm um} & g_{\rm back} \end{pmatrix} \begin{pmatrix} f_1 \\ f_2 \end{pmatrix}, \tag{S4}$$

where $N(0)$ is the density-of-states at the Fermi level. As we explain in the main text, both $g_{\rm back}$ and $g_{\rm um}$ are positive. Thus, the largest eigenvalue and the eigenvector are

$\lambda \sim N(0)(g_{\rm back}+g_{\rm um})$ and $\boldsymbol{f}=(1,1)$, respectively. After the Fourier transformation, the real-space form factor in the triangular lattice model in Fig. S1 is given as $\delta t_{CA}(R\boldsymbol{e}_\perp) \sim f_{CA}(\boldsymbol{k}_{\rm A})e^{i\pi R}$ and $\delta t_{AC}(R\boldsymbol{e}_\perp) \sim f_{AC}(\boldsymbol{k}_{\rm C})e^{i\pi R}$. Here, R is an integer, and $\boldsymbol{e}_\perp$ is a unit vector perpendicular to $\boldsymbol{q}_3$. By making comparison between Fig. S1 and Fig. 1 (b) in the main text, the nearest-neighbor hopping modulations in Fig. S4 (a) are given as $(\delta t, \delta t') = (\delta t_{CA}(\boldsymbol{0}), \delta t_{AC}(\boldsymbol{e}_\perp)) \propto (1,-1)$.

The same bond-order in the kagome lattice model is shown in Fig. S4 (b). When $\delta t = -\delta t'$, it is equivalent to Fig. 2 (d) in the main text. Therefore, the essential origin of the bond-order is naturally understood based on a simple two vHS model in Eq. (S4).

C: Unfolded Fermi surface under triple-$\boldsymbol{q}$ state

In the triple-$\boldsymbol{q}$ DW state, both the FS and the bandstructure are folded into the folded BZ. They can be unfolded into the original size BZ, which correspond to the ARPES measurement in the DW state. The obtained unfolded FS in the case of $\max_{\boldsymbol{k}}\{f_{\boldsymbol{q}_n}(\boldsymbol{k})\} = 0.018$ [eV] is shown in Fig. S5. Here, the spectra around the vHS points are gapped. This result is consistent with the recent ARPES studies.

D: GL free energy in D_{6h} kagome model

Here, we briefly review the GL free energy of a D_{6h} system up to the fourth order and explain that the triple-$\boldsymbol{q}$ order is stabilized by the third-order term. We introduce three real order parameters ϕ_n ($n=1-3$) and express the bond-order functions as $\phi_n \hat{f}_{\boldsymbol{q}_n}(k)$, where $\hat{f}_{\boldsymbol{q}_n}(k)$ is the normalized dimensionless form factor given by the linearized DW equation. [Note that the phase factor of the form factor is fixed by the Hermite condition $f_q^{lm}(k) = [f_{-q}^{im}(k+q)]^*$.] The GL free energy is given as [48]

$$
\begin{aligned}
F &= a[\phi_1^2+\phi_2^2+\phi_3^2] + b\phi_1\phi_2\phi_3 \\
&\quad + c[\phi_1^4+\phi_2^4+\phi_3^4] + d[\phi_1^2\phi_2^2+\phi_2^2\phi_3^2+\phi_3^2\phi_1^2]
\end{aligned} \tag{S5}
$$

where the second-order coefficient a is proportional to $1-\lambda_{\rm bond}$. The fourth-order coefficients c, d are positive. Here, the third-order coefficient b is nonzero because of the momentum conservation relation $\boldsymbol{q}_1+\boldsymbol{q}_2+\boldsymbol{q}_3=\boldsymbol{0}$. Note that the sign of b is reversed under the transformation $f \to -f$.

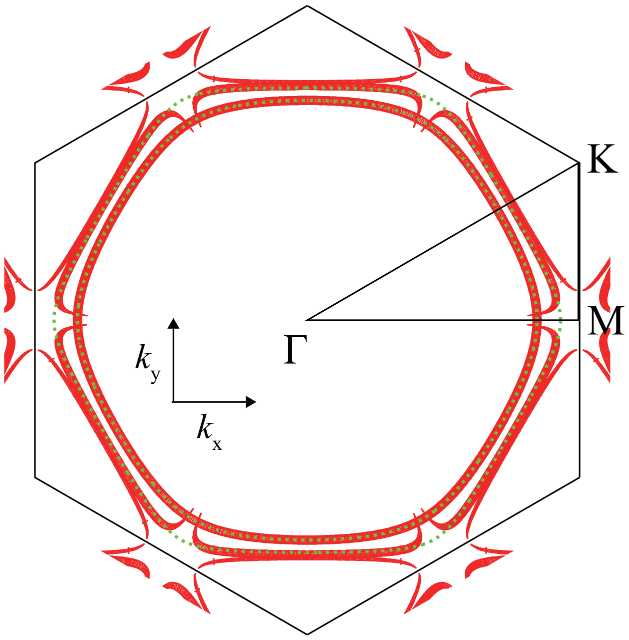

FIG. S5: **Unfolded FS under the triple-$\boldsymbol{q}$ bond-order:** The FS near the vHS points is reconstructed by the bond-order. The result for $\max_{\boldsymbol{k}}\{f_{\boldsymbol{q}_n}(\boldsymbol{k})\} = 0.018$ [eV] is shown. The green dotted lines represent the original FS.

One can calculate the coefficients b, c, and d microscopically based on their diagrammatic expressions given in our previous paper on 1T-TaS_2 [48]: The coefficient b is given by the triangle diagram composed of three Green functions and three form factors. In a similar way, the coefficients c and d are given by the square diagrams.

Based on the GL free energy, the single-$\boldsymbol{q}$ solution $(\phi_1, \phi_2, \phi_3) = (\phi, 0, 0)$ occurs when $a \leq 0$ as the second-order transition. On the other hand, in the case of the triple-$\boldsymbol{q}$ order, the free energy is $F(\phi) = a\phi^2 + b\phi^3/3\sqrt{3} + (c+d)\phi^4/3$. In the case $a > 0$, its local minimum is given at $\phi_0 = \dfrac{-\sqrt{3}b}{8(c+d)}\left(1 - \sqrt{1 - \dfrac{32a}{b^2}(c+d)}\right)$, which is positive [negative] for $b < 0$ [$b > 0$]. The free energy $F(\phi_0)$ becomes negative when $a < b^2/[36(c+d)]$ (=positive). Therefore, triple-$\boldsymbol{q}$ order is realized as the first order transition when a is positive. (That is, ϕ jumps from zero to ϕ_0 at finite $a > 0$.) Consistently with this analysis, the (inverse) star of David bond-order emerges as a weak first order transition experimentally.

E: Derivation of SC gap equation

Here, we discuss the reason why bond-order fluctuations mediate the pairing interaction. In Ref. [5], the authors studied the orbital fluctuation mediated s-wave superconductivity in Fe-based superconductors. In that study, the electron-boson coupling (=form factor) is an orbital-dependent but $\boldsymbol{k}$-independent charge quadrupole operator: $\hat{f}^{\boldsymbol{q}}(\boldsymbol{k}) = \hat{O}_\Gamma$ ($\Gamma = xz, yz, xy$). In the main text, we obtain the development of bond-order fluctuations with the $\boldsymbol{k}$-dependent form factor in AV_3Sb_5, which is given by the inter-sublattice vertex corrections (VCs) that are dropped in the RPA. We reveal that bond-order fluctuations mediate significant "beyond-Migdal" pairing interaction thanks to the $\boldsymbol{k}$-dependent form factor [44], and therefore s-wave and p-wave SC states emerge in AV_3Sb_5.

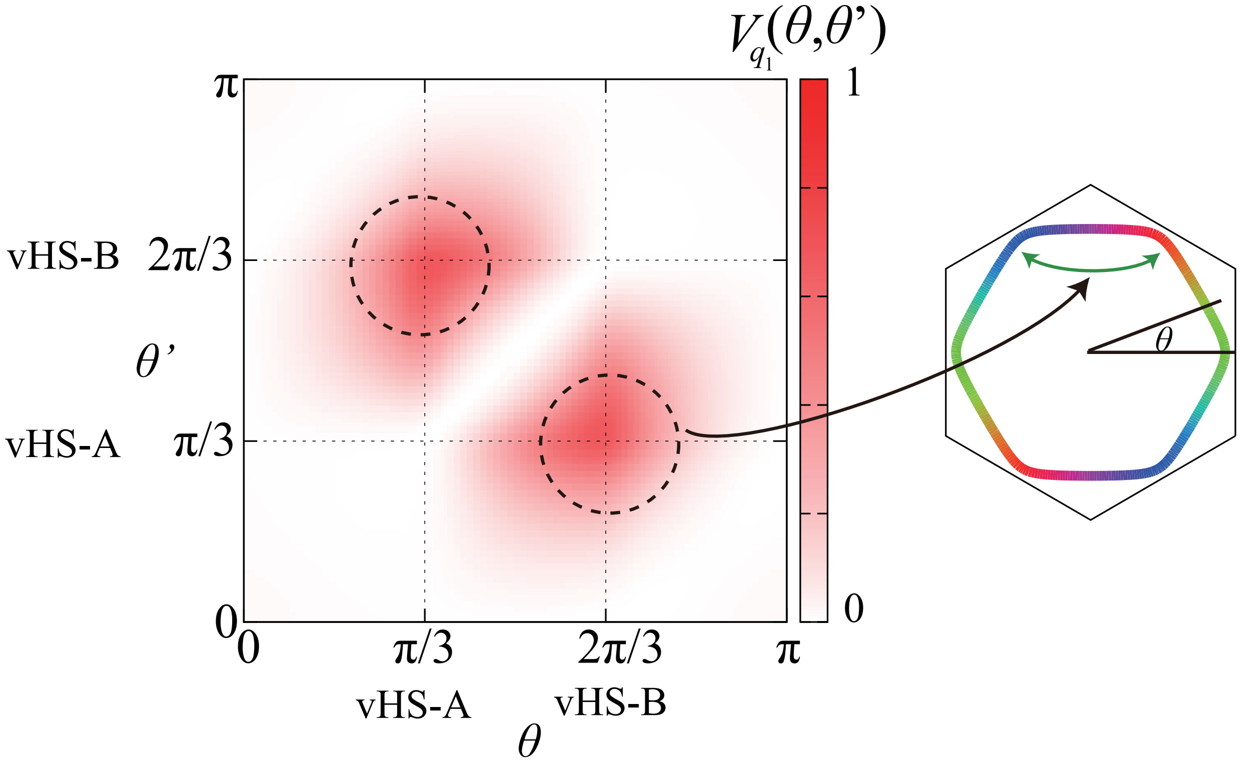

FIG. S6: **Pairing interaction on b_{3g}-orbital FS due to bond-order fluctuations:** We present $V_1(\theta, \theta')$ due to $\boldsymbol{q} \approx \boldsymbol{q}_1$ given in (S10) in the case of $\bar{I}_{\boldsymbol{q}} = 1$ and $\lambda_{\rm bond} = 0$. Here, $\theta = \arctan(k_y/k_x)$ and $\theta' = \arctan(k'_y/k'_x)$.

In the following, we discuss the pairing interaction due to the bond-order fluctuations in kagome metal by following Ref. [44]. Hereafter, we drop the orbital indices just to simplify

the notation. The pairing interaction between Cooper pairs $(k, -k)$ and $(k', -k')$ due to charge-channel full four-point vertex in Fig. 7 (c) is given as $V(k, k') \propto \Gamma^c_{k'-k}(k, -k')$. We derive a convenient simple expression of $\Gamma^c_{\boldsymbol{q}}(k, k')$, we introduce the following approximation for the kernel function:

$$I_{\boldsymbol{q}}(\boldsymbol{k}.\boldsymbol{k}') = \bar{I}_{\boldsymbol{q}} f_{\boldsymbol{q}}(k) f^*_{\boldsymbol{q}}(k'), \tag{S6}$$

where $f_{\boldsymbol{q}}(k)$ is the form factor for the largest eigenvalue of the DW equation. By inserting Eq. (S6) into the DW equation, the eigenvalue is given as $\lambda_{\boldsymbol{q}} = \bar{I}_{\boldsymbol{q}} \chi^0_f(\boldsymbol{q})$, where

$$\chi^0_f(\boldsymbol{q}) = -\frac{T}{N} \sum_k G(k+\boldsymbol{q}) G(k) \tilde{f}_{\boldsymbol{q}}(k) \tilde{f}^*_{\boldsymbol{q}}(k). \ \ (> 0) \tag{S7}$$

Then, the full four-point vertex is given as

$$\Gamma^c_{\boldsymbol{q}}(k, k') = \frac{\bar{I}_{\boldsymbol{q}} f_{\boldsymbol{q}}(k) f^*_{\boldsymbol{q}}(k')}{1 - \lambda_{\boldsymbol{q}}}. \tag{S8}$$

Therefore, the pairing interaction is $V(k, k') = \frac{\bar{I}_q f_{\boldsymbol{q}}(k) f^*_{\boldsymbol{q}}(-k')}{1-\lambda_{\boldsymbol{q}}}$, where $\boldsymbol{q} = \boldsymbol{k}' - \boldsymbol{k}$. Note that the relation $f^*_{\boldsymbol{q}}(-k') = f_{-\boldsymbol{q}}(-k)$ holds for $\boldsymbol{q} = \boldsymbol{k}' - \boldsymbol{k}$ due to the Hermite condition of the form factor.

Considering that the $\boldsymbol{q}$-dependence of the form factor is moderate, the total pairing interaction due to triple-$\boldsymbol{q}$ bond-order fluctuations is approximately given as

$$V_{\rm bond}(k, k') \ = \ \frac{1}{2} \sum_n^{1,2,3} V_n(k, k'), \tag{S9}$$

$$\begin{aligned} V_n(k, k') \ &= \ \frac{\bar{I}_{\boldsymbol{q}} \bar{f}_{\boldsymbol{q}_n}(\boldsymbol{k}) \bar{f}^*_{\boldsymbol{q}_n}(-\boldsymbol{k}')}{1 - \lambda_{\boldsymbol{k}'-\boldsymbol{k}}} \\ &\approx \ \frac{\bar{I}_{\rm bond} \bar{f}_{\boldsymbol{q}_n}(\boldsymbol{k}) \bar{f}_{-\boldsymbol{q}_n}(-\boldsymbol{k})}{1 - \lambda_{\rm bond}} \frac{1}{1 + \xi^2(\boldsymbol{q}_n - (\boldsymbol{k}' - \boldsymbol{k}))^2}, \end{aligned} \tag{S10}$$

where $\bar{f}^{\boldsymbol{q}}(\boldsymbol{k}) \equiv \sum_{l,m} \tilde{f}^{\boldsymbol{q}}_{lm}(\boldsymbol{k}) u_{l,b}(\boldsymbol{k}+\boldsymbol{q})^* u_{m,b}(\boldsymbol{k})$. Here, $u_{l,b}(\boldsymbol{k}) = \langle l, \boldsymbol{k} | b, \boldsymbol{k} \rangle$ is the unitary transformation matrix element between orbital l and conduction band b, and $\tilde{f}^{\boldsymbol{q}}(\boldsymbol{k}) \equiv (f^{\boldsymbol{q}}(\boldsymbol{k}, \pi T) + f^{\boldsymbol{q}}(\boldsymbol{k}, -\pi T))/2$. We also approximate $\lambda_{\boldsymbol{q}} \approx \lambda_{\rm bond} - b(\boldsymbol{q} - \boldsymbol{q}_n)^2$ with $b \approx \xi^2(1 - \lambda_{\rm bond})$ for $\boldsymbol{q} \sim \boldsymbol{q}_n$. Here, we set $\bar{f}^{BA}_{\boldsymbol{q}_1}(k_{\rm B}) = 1$. Then, the coupling constant $\bar{I}_{\rm bond}$ is directly given by $g_{\rm um}$ that is obtained in the main text. We stress that $\bar{f}_{\boldsymbol{q}_n}(k) \bar{f}_{-\boldsymbol{q}_n}(-k)$ is

positive for even-parity bond-order. In the main text, we set $\bar{I}_{\rm bond} = g_{\rm um}\,|\bar{f}_{\boldsymbol{q}_1}(\boldsymbol{k}_{\rm A})| = 1$ in the pairing interaction in Eq. (S10).

Figure S6 is the pairing interaction $V_1(\theta,\theta')$ on the b_{3g}-orbital FS due to the bond-order fluctuations at wavevector $\boldsymbol{q} \approx \boldsymbol{q}_1$. by setting $\bar{I}_{\boldsymbol{q}} = 1$, $\lambda_{\rm bond} = 0$ and $\xi = 0$. Here, $\theta = \arctan(k_y/k_x)$ and $\theta' = \arctan(k'_y/k'_x)$. Thus, strong attractive pairing interaction is induced by the bond-order fluctuations around the vHS points. This is the driving force of the s-wave and p-wave pairing states obtained in the main text.

In the main text, we solve the gap equation in the presence of bond and spin fluctuations.

$$V_{\rm s}^{\rm SC}(k,k') \;=\; V_{\rm bond}(k,k') - \frac{3}{2}U^2\chi^s(k-k') - U, \tag{S11}$$

$$V_{\rm t}^{\rm SC}(k,k') \;=\; V_{\rm bond}(k,k') + \frac{1}{2}U^2\chi^s(k-k'), \tag{S12}$$

where s (t) represents the singlet (triplet) pairing interaction. The diagrammatic expression of the gap equation due to $V_{\rm bond}(k,k')$ is depicted in Fig. 4 (a) in the main text. In solving the gap equation, we set the BCS cutoff energy ω_c for $V_{\rm bond}$ because the energy-scale of bond-order fluctuations is much smaller than E_F. Here, we set $\omega_c = 0.02$. Note that the pairing interaction for the band b, b' can be derived from that in the orbital representation by using the unitary transformation matrix $u_{l,b}(\boldsymbol{k})$.

It is noteworthy that the present bond-order fluctuating pairing mechanism is outside of the Migdal approximation, in which the form factor is assumed to be $\boldsymbol{k}$-independent. The present bond-order fluctuating mechanism has a close similarity to the multipole-fluctuation pairing mechanism developed in Refs. [43, 44].

F: Comparison between the present DW equation theory and mean-field theory

A great merit of the present paramagnon-interference theory is that the star of David bond-order is naturally obtained based on a simple Hubbard model without introducing any off-site Coulomb interactions. Here, we briefly review the results of the mean-field theory based on the U-V Hubbard model, where V is the nearest-neighbor Coulomb interaction. For this purpose, we solve the linearized mean-field equation with the optimized form factor, which is given by Eq. (2) with the Hartree-Fock kernel function made of U and V. Figure S7 (a) shows the obtained eigenvalues as a function of V/U at $U = 0.79$. (Here, we drop b_{2g} orbitals in the kagome metal Hubbard model introduced in the main text, because b_{2g}

orbitals are not essential for the bond-order.) The spin-density-wave (SDW) instability (with spin form factor $f^s = 1$) is the largest for $V \lesssim 0.4U$. The SDW is replaced with the spin-bond-order (spin-BO), $f^s \neq 1$) for $0.4U \lesssim V \lesssim 0.6U$, and the charge-density-wave (CDW) instability (with charge form factor $f^c = 1$) is the largest for $V \gtrsim 0.6U$. Figure S7 (b) shows the results as functions of U/V at $V = 0.32$. Thus, the charge bond-order (charge-BO) instability is secondary in both Figs. S7 (a) and (b).

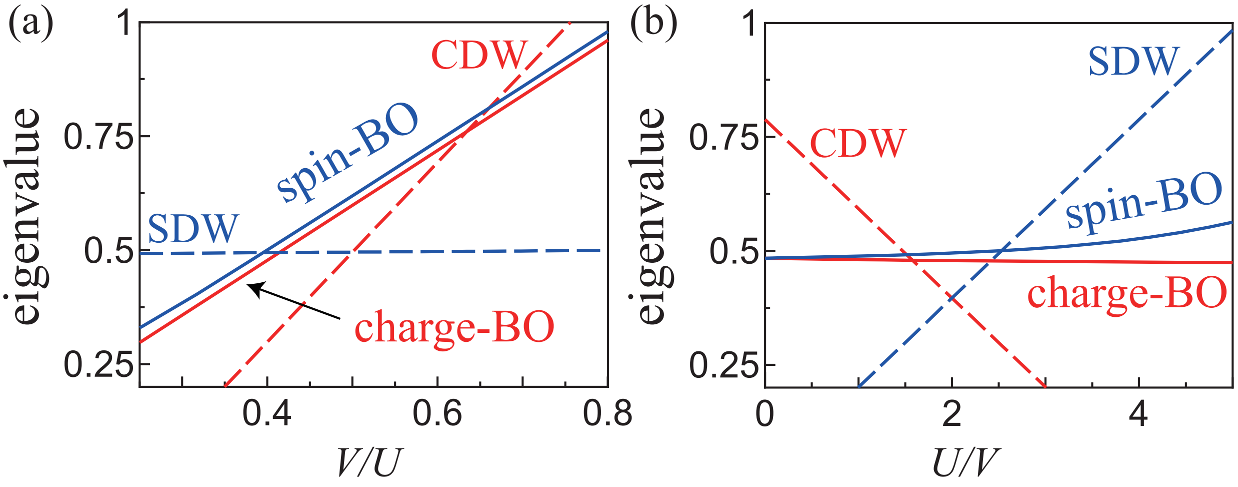

FIG. S7: **Mean-field analysis for U-V Hubbard model**: (a) Eigenvalues of the mean-field density-wave equation as a function of V/U at $U = 0.79$, and (b) those as a function of U/V at $V = 0.32$. Here, the charge-BO instability is smaller than other instabilities.

In contrast, the charge bond-order instability is solely magnified in the DW equation with MT and AL terms even for $V = 0$. Figure S8 exhibits the spin-channel and charge-channel eigenvalues, $\lambda^s_{\boldsymbol{q}}$ and $\lambda^c_{\boldsymbol{q}}$, in the DW equation at $\boldsymbol{q} = \boldsymbol{0}$ and $\boldsymbol{q} = \boldsymbol{q}_1$ [63]. We see that $\lambda^c_{\boldsymbol{q}=\boldsymbol{q}_1}$, which is equivalent to λ_{bond} in the main text, solely increases with increasing α_S ($\propto U$). Thus, the bond-order solution is obtained for wide parameter range. Interestingly, $\lambda_{\boldsymbol{q}=\boldsymbol{0},\text{bond}} \equiv \lambda^c_{\boldsymbol{q}=\boldsymbol{0}}$, which corresponds to A_{1g} bond-order with sign-reversing form factor, starts to develop for $\alpha_S \gtrsim 0.8$.

By solving the spin-channel DW equation [63], we reveal that the spin channel eigenvalue $\lambda_{\text{spin}} \equiv \max_{\boldsymbol{q}} \lambda^s_{\boldsymbol{q}}$ is smaller than α_S only slightly, and it corresponds to the SDW solution with $f^s \approx 1$. (Note that $\lambda_{\text{spin}} = \alpha_S$ in the mean-field approximation for $V = 0$.) We stress that the spin-BO ($f^s \neq 1$) instability is always smaller than the SDW instability. In the present theory, the charge-channel AL term is proportional to the convolution $C^c(\boldsymbol{q}_1) = \sum_{\boldsymbol{k}} \chi^s(\boldsymbol{k} +$

$\boldsymbol{q}_1)\chi^s(\boldsymbol{k})$, while the spin-channel one is proportional to $C^s(\boldsymbol{q}_1) = \sum_{\boldsymbol{k}} \chi^s(\boldsymbol{k}+\boldsymbol{q}_1)\chi^c(\boldsymbol{k})$, as we discussed in Refs. [9, 63]. Since $C^c(\boldsymbol{q}_1) \gg |C^s(\boldsymbol{q}_1)|$ for $\alpha_S \gtrsim 0.75$, only the charge bond-order eigenvalue $\lambda_{\rm bond}$ is strongly enlarged and exceeds unity in Fig. S8. Thus, the instability of the smectic bond-order is robust in kagome metals, while any spin-channel eigenvalues are smaller than unity even if the quantum interference mechanism is taken into account. This is a great merit of the present DW equation analysis.

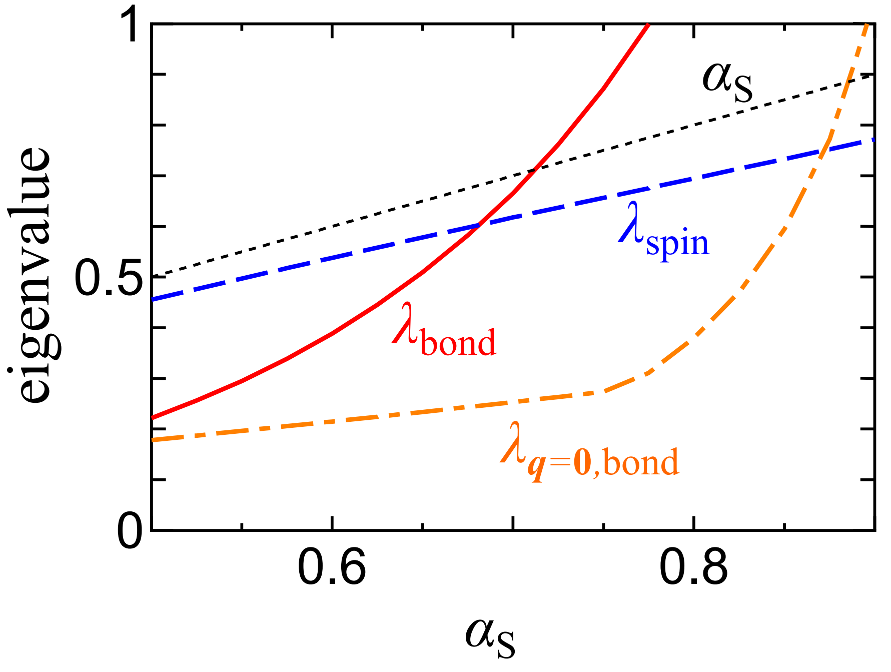

FIG. S8: **DW equation analysis for U Hubbard model**: Eigenvalue of DW equation with MT and AL terms as a function of α_S ($\propto U$). Here, the eigenvalue of charge-channel bond-order $\lambda_{\rm bond} \equiv \lambda^c_{\boldsymbol{q}_1}$ solely increases due to the AL terms. In contrast, the spin-channel eigenvalue $\lambda_{\rm spin} \equiv \lambda^s_{\boldsymbol{q}_1}$ remains small even if the spin-channel vertex corrections are taken into account. Interestingly, $\lambda_{\boldsymbol{q}=\boldsymbol{0},\rm bond} \equiv \lambda^c_{\boldsymbol{q}=\boldsymbol{0}}$, which corresponds to A_{1g} bond-order with sign-reversing form factor, starts to develop for $\alpha_S \gtrsim 0.8$.

G: Realistic model Hamiltonian based on the first-principles study

Based on the first-principles study, we derive the two-dimensional realistic model for CsV_3Sb_5, which we analyzed in the main text. First, we perform the Wien2k DFT calculation of CsV_3Sb_5 under pressure P [GPa], using the crystal structure data in Ref. [58]. Next,

we derive the 30 orbital (15 3d-orbitals + 15 5p-orbitals) tight-binding model, $H_0^{\rm DFT}(P)$, using the Wannier90 software. The DFT band dispersions are fitted almost perfectly. Here, we drop the inter-layer hopping integrals. The bandstructure along the k_x-axis at $P=0$ is shown in Fig. S9 (a). The green line is the b_{3g}-orbital band, and its width represents the b_{3g}-orbital weight. The bandstructure in Fig. S9 (a) is qualitatively similar to ARPES data. However, Fig. S9 (a) is different from the experimental CsV_3Sb_5 bandstructure [31, 53, 54] at the following two points: (i) The vHS energy $E_{\rm vHS}$ is not adjacent to the Fermi level $E_{\rm F}$. (ii) The b_{3g}-band crosses a 5p-band around M point, so the b_{3g}-orbital weight disappears via the band hybridization except on the k_x-axis.

We will fix these discrepancies by introducing *minimum changes* in the Hamiltonian. Here, we shift 15 5p-orbital levels by $\Delta E_p = -0.2$ [eV] and introduce the hole-doping by 0.1 to make $E_{\rm vHS}$ closer to $E_{\rm F}$. (The orbital-dependent energy shift method is frequently applied in Fe-based superconductors to reproduce experimental FSs [9].) The obtained modified bandstructure is shown in Fig. S9 (b) and its FS is presented in Fig. 6 (a), which are consistent with recent STM and ARPES measurements [31, 53, 54]. We use this model as the model Hamiltonian at ambient pressure, $H_0(P=0)$.

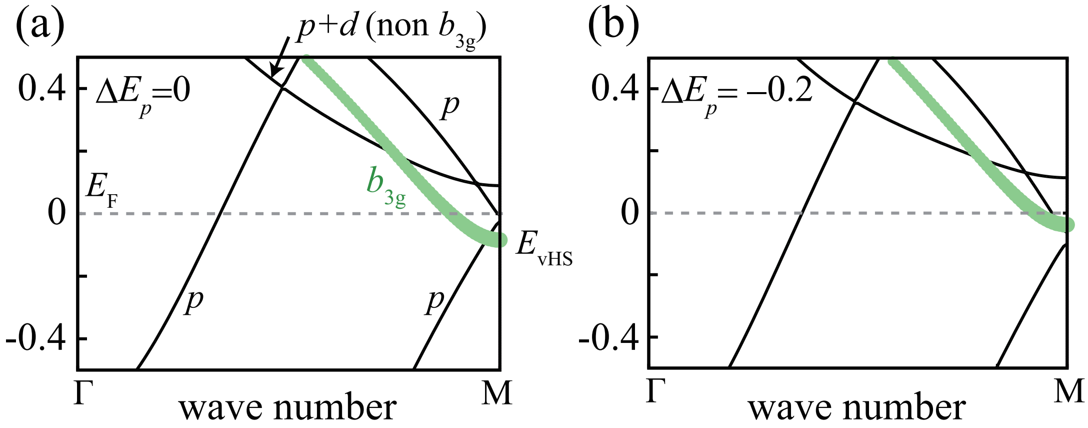

FIG. S9: **Band structure based on the first-principles calculation**: (a) Original DFT band structure for CsV_3Sb_5 in the case of $\Delta E_p = 0$. (b) Modified band structure with $\Delta E_p = -0.2$ and 0.1 hole-doping.

Next, we discuss the pressure dependence of the Hamiltonian caused by the systematic change in the crystal structure. It is described by the "pressure Hamiltonian $\Delta H_0^{\rm DFT}(P) \equiv$

$H_0^{\rm DFT}(P) - H_0^{\rm DFT}(0)$". Then, the model Hamiltonian is given as

$$H_0(P) = H_0(0) + \Delta H_0^{\rm DFT}(P). \tag{S13}$$

This method has been successfully applied to the study of P-T phase diagram in Fe-based superconductors. The obtained FSs at $P = 0$ and 3GPa are shown in Figs. 6 (a) and (b). Under pressure, the volume of b_{3g}-orbital FS is reduced because the b_{3g}-orbital level shifts downward relatively. The self-doping on the b_{3g}-FS ($\sim$ 1.5% at P = 3GPa) derived from $\Delta H_0^{\rm DFT}(P)$ will be reliable. At the same time, the bandwidth increases under pressure, and the spin Stoner factor α_S is reduced by 0.03 at P = 3GPa. (In this model, $\alpha_S = 0.95$ at $U = 2.7$ [eV] when $P = 0$.) By using Eq. (S13), the pressure-induced C-IC bond-order transition is explained in the main text. It is an important future problem to analyze three-dimensional Hubbard model of CsV_3Sb_5.
